# Sustainable synthesis and advanced optimization of *Prosopis juliflora* biomass catalyst for efficient biodiesel production and environmental impact assessment

**DOI:** 10.1038/s41598-025-88355-z

**Published:** 2025-02-06

**Authors:** Thota S S Bhaskara Rao, Manikandaraja Gnanaprakasam, Rajayokkiam Manimaran, Dhinesh Balasubramanian, Utku Kale, Artūras Kilikevičius

**Affiliations:** 1 Department of Mechanical Engineering, Madanapalle Institute of Technology & Science, Madanapalle, Andhra Pradesh India; 2 School of Mechanical Engineering, SRM Institute of Science and Technology, Tiruchirappalli Campus, Tiruchirappalli, Tamil Nadu India; 3 Department of Mechanical Engineering, Mepco Schlenk Engineering College, Sivakasi, Tamil Nadu India; 4 Department of Aeronautics, and Naval Architecture, Faculty of Transportation Engineering and Vehicle Engineering, Budapest University of Technology and Economics, Budapest, Hungary; 5 Mechanical Science Institute, Vilnius Gediminas Technical University, Plytinės g. 25, Vilnius, 10105 Lithuania

**Keywords:** PJBC catalyst, WTSB biodiesel, RSM-GA optimization, DI diesel engine, Environmental effects, Energy science and technology, Engineering

## Abstract

The present research focuses on developing an innovative biochar-based heterogeneous catalyst from *Prosopis Juliflora* biomass waste using response surface methodology and genetic algorithm (GA) to optimize pyrolysis parameters, achieving a 46.31% PJBC yield from 60 mg of biomass at 790 °C for 60 min. The pyrolyzed PJBC is characterized using SEM, FTIR, XRD, EDX, BET, XPS analyses, and physico-chemical measurements to confirm its catalytic activity. Now, the newly synthesized PJBC serves as an efficient catalyst for waste *Trichosanthes cucumerina* seed biodiesel (WTSB) production from waste *Trichosanthes cucumerina* seed bio-oil through trans-esterification, achieving a maximum yield of 97.42%. Also, the WTSB exhibits excellent physico-chemical properties that meet most of the ASTM D6751 standards for biodiesel and closely align with the characteristics of conventional diesel fuel. Therefore, this research utilized neat WTSB and WTSB/diesel blends (WTSB25, WTSB50, and WTSB75) in a direct injection (DI) diesel engine at variable load settings. Among all WTSB blends, the WTSB25 blend showed closer variations of 1.65% lower BTE and 9.29% higher BSEC when compared to conventional diesel fuel readings. Its peak in-cylinder pressure and heat release rate were similar to those of diesel fuel at 100% engine load. Emission analysis indicated that the WTSB25 reduced specific HC, CO, and smoke opacity emissions by 8.39%, 13.97%, and 4.18%, respectively. However, specific NO emissions increased slightly by 3.05% compared to diesel fuel. Thus, WTSB25 is validated as a viable diesel alternative requiring no significant engine modifications. The environmental impact, lifecycle and economic feasibility are also discussed.

## Introduction

In the twenty-first century, energy plays a crucial role in shaping nations’ social and cultural development worldwide. As energy demand rises, along with environmental pollution and the depletion of fossil fuel reserves, the fuel industry actively seeks sustainable alternatives to diesel fuel used in direct ignition (DI) diesel engines^1^. The global energy-related emissions are predicted by the International Energy Agency (IEA) to rise from 32.3 billion metric tonnes in 2012 to 35.6 billion metric tonnes in 2020 and then to 43.2 billion metric tonnes by 2040. By overcoming the need for alternative fuels and the rise in global emissions, despite modest production quantities, first-generation biofuels derived from agricultural crops of maize directly replace petroleum and diesel^2^. On the other hand, second-generation biofuels, which come from waste materials used in buildings and cities, forest leftovers, and energy crops, are more advantageous since they lower carbon emissions, increase energy efficiency, and lessen the need for fossil fuels. The necessity for these cutting-edge biofuels is highlighted by the expanding problem of urban solid waste and the reliance on non-renewable energy for transportation^3^. Waste from municipal solid sources pollutes water supplies, damages the environment, and is expensive. Every year, approximately 931 million metric tons of basic crops are lost, accounting for about 10% of the total emissions of greenhouse gases^4^. To minimize pollution and promote economic growth, Vision 2030 seeks to improve environmentally friendly waste-to-energy through the advancement of technology. Making biodiesel out of garbage *Trichosanthes cucumerina* biomass has the potential to lower pollution and accelerate economic growth. Transesterification is the most practical and well-established commercial method for making methyl esters^5^. Various homogeneous catalysts, such as NaOH, KOH, and H_2_SO_4_, are frequently employed in this method to produce industrial biodiesel. However, there are disadvantages to homogeneous catalysts, such as high operating expenses and negative environmental effects^6^. In order to solve these problems, researchers are looking toward reusable and insoluble heterogeneous catalysts. Significantly, green heterogeneous catalysts from wood biomass increase the production of biodiesel because the materials are safer for the environment, reusable, and less hazardous^7^. According to estimates, India has produced around 11 tons of wood biomass waste per hectare on average over the last three years from *Prosopis juliflora*, with Tamil Nadu accounting for between 40% and 50% of this total^8^. The biomass of *Prosopis juliflora* burns continuously and fast, with a high caloric value attributed to its high lignin and carbon content. It is a well-liked bioenergy source because of its low hydration and ash content. So, for producing sustainable biochar from *Prosopis juliflora* biomass waste can also be used as a catalyst for biodiesel production^9^. Kang et al.^10^ investigated the many uses of biochar from biomass waste in fuels, specialty materials, chemical industries (catalysts, adsorption agents, and sewage treatment), farming, credits for carbon emissions, and other fields of study. In-depth research has been done on the use of biochar as a catalyst to convert biomass into biofuels with the right modifications, activation, and integration via knowledge of its physical chemistry. It was proven that biochar-based catalysts provide higher yields while producing biodiesel than catalysts generated chemically by Foroutan et al.^11^. In addition, Table 1 lists the availability of a range of waste biomasses, unique production methods, and optimized process parameters tailored to maximize biochar production from different crops and their respective uses to support the current research.

**Fig. 1:**
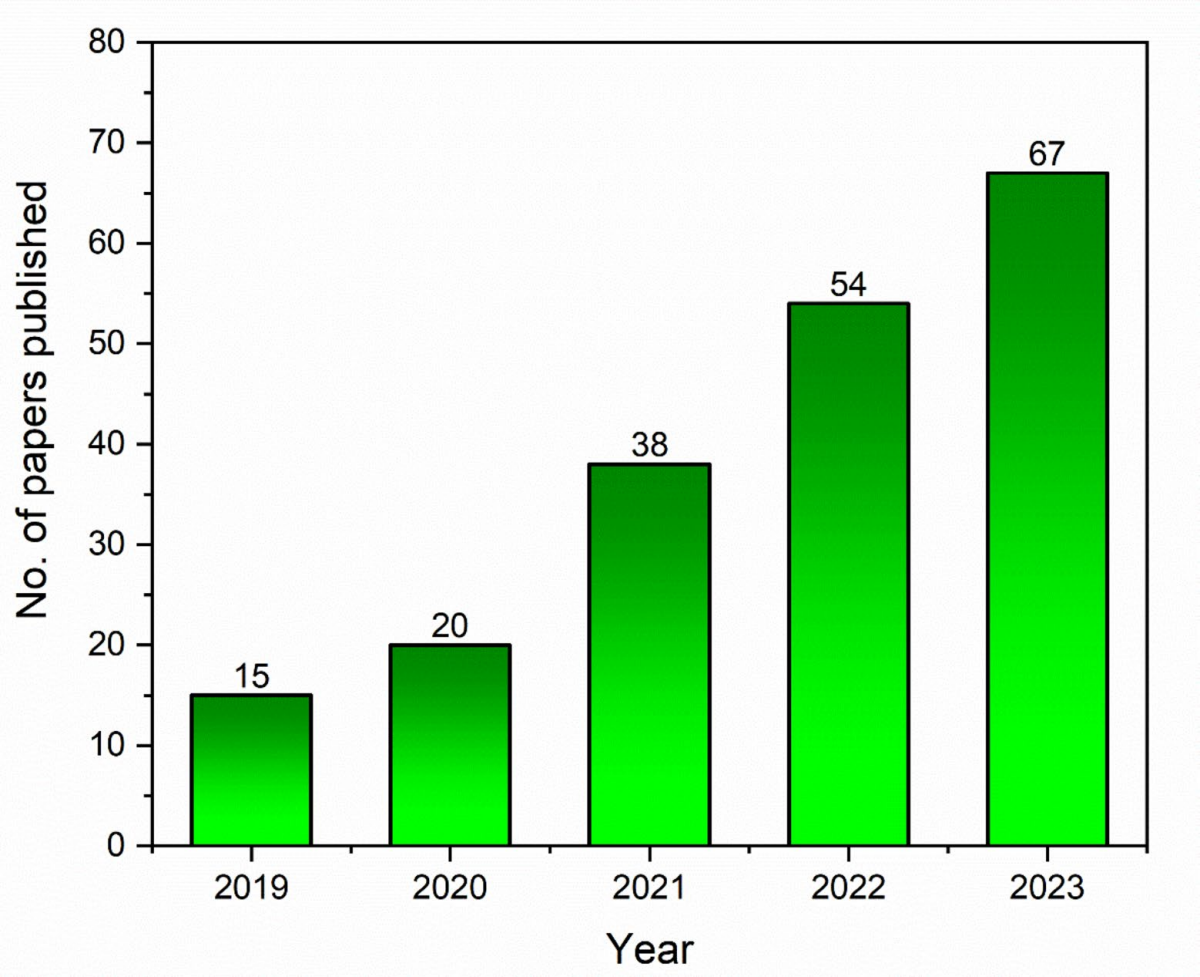
Number of papers published on biochar and its applications (data collected from the database of ScienceDirect).

The volume of research that has been published on biochar synthesis, characterization, and its applications since 2019 is represented in Fig. 1. It was discovered that the volume of documents published between 2019 and 2023 on biochar-based catalysts for different applications increased significantly. The sheer number of articles available ensured that researchers have recently paid more attention to using biochar-based catalysts for biofuel production from different biomasses and evaluated their applications.

**Table 1:** Feedstock, production technique, optimized process parameters on higher biochar yield from various crops with their suitable applications.

Feedstock	Production technique	Optimum process parameters	Biochar yield (wt%)	Application	Reference
Rice husk	Calcination process	Heating temperature (450 °C) and Process duration (180 min)	60	Catalytic activity	^12^
Corncob	Fixed bed pyrolysis	Temperature (550 °C) and Heating time duration (15 min)	30.42	Soil amendment	^13^
Canola straw	Fixed bed pyrolysis	Reaction temperature (400 °C) and Heating duration (120 min)	46.30	Lead adsorption	^14^
Peanut shell	Calcination process	Heating temperature (450 °C) and duration (240 min)	41	Acting as a catalyst for biodiesel production	^15^
Wheat straw	Fixed bed pyrolysis	Pretreatment at 500 °C and 15 min time duration	30.42	Soil amendment and Wastewater treatment	^16^
Rice straw	Fixed bed pyrolysis	Heating temperature (500 °C) and its time duration (120 min)	46.31	Ammonia adsorption	^17^
Corn stover	Microwave-assisted pyrolysis	Microwave treatment from temperature 450 to 550 °C with 20 min operating time.	29	Mitigation of soil N_2_O emissions and catalytic application	^18^
Pine wood sawdust	Microwave pyrolysis	Pyrolysis temperature (470 °C) and total process time (12 min)	17	Biosorbent for PAHs removal from wastewater	^19^
Coconut husk	Wet pyrolysis	Hydrothermal carbonization temperature (140 °C) and heating time (240 min)	76	Soil amendment, carbon sequestration and anaerobic digestate application	^20^

Chutia and Phukan^21^ used leftover *mesua assamica* seed shells to create the novel heterogeneous catalyst Fe_3_O_4_@biochar@SO_3_H for the production of cost-effective biodiesel from various oil feedstocks. Fourier transform infrared (FTIR), X-ray photoelectron spectroscopy (XPS), and field emission transmission electron microscopy (FETEM) determine the presence of –SO_3_H in heterogeneous catalyst and their mean diameter and surface area. This study concluded that the synthesized Fe_3_O_4_ @biochar@SO_3_H catalyst produces maximum biodiesel yield from *mesua assamica* seed oil (96.80%), jatropha oil (95.30%) and soybean oil (95.80%) under optimized operating parameters. The calcined filter cake (CFC) heterogeneous catalyst was synthesized from the waste filter cake of a sugar beet processing plant, producing rapeseed biodiesel. Initially, the presence of CaO@CFC heterogeneous catalyst is ensured through various characterization tests, which confirm the production of methyl ester. The CaO@CFC gives a maximum 97.90% rapeseed biodiesel conversion rate at ideal conditions of 60 °C melting point, 9:1 methanol/rapeseed bio-oil molar concentration ratio, 10% CFC heterogeneous catalyst, and 60 min duration than other homogeneous type catalysts^22^. Similarly, Oyekunle et al.^23^ reviewed various feedstocks, their properties, the use of heterogeneous catalysts, optimization of process parameters, and catalytic activity in the transesterification process for biodiesel production from different biomass wastes. This study confirms that solid-based heterogeneous catalysts’ basicity and textural properties significantly influence biodiesel yield. These catalysts are preferred due to their reusability and ease of product separation.

Response Surface Methodology (RSM) is a statistical and mathematical approach used to model and optimize output responses influenced by multiple input variables. It identifies optimal responses by adjusting process parameters within defined constraints^24^. According to the literature, Wang^25^ produced biodiesel from spent coffee grounds oil using a novel activated carbon green catalyst derived from pomegranate and orange peels (POPAC). Various analytical methods ensure the POPAC catalytic activity. The highest yield of 95.24% was achieved with POPAC catalyst under optimal conditions: a molar proportion of 11.02, green catalyst of 2.56 wt%, ultrasonic time of 30.77 min, and a temperature of 60 °C using RSM. Due to their exceptional catalytic performance, significant recyclability, and eco-friendliness, POPAC green catalysts are highly recommended for biodiesel production. The performance of a diesel engine running on biodiesel from spent coffee grounds oil (SCGOB) and SCGOB/diesel blends showed improved performance metrics and reduced tailpipe emissions than using neat diesel. Dubey et al.^26^ optimized the process parameters for the transesterification of waste cooking oil (WCO) into biodiesel. A maximum WCO biodiesel yield of 92% was achieved at a reaction temperature of 60 °C, with 0.75 wt% catalyst, 80 min reaction time, and a 5:1 methanol-to-oil molar ratio using the RSM technique.

Sun et al.^27^ investigated the use of Fe_3_O_4_@UiO-66-NH_2_ catalyst for synthesizing biodiesel from restaurant waste oil and analyzed its effects on diesel engine characteristics. The study achieved a maximum biodiesel efficiency of 98.72% under optimal conditions with the RSM approach, which included a CH_3_OH to waste oil ratio of 11.38:1, a reaction period of 8.84 min, a catalyst concentration of 3.27%, and a mixing speed of 723.38 rpm. Several techniques, such as FTIR, BET, EDX and XRD, were used to characterize the Fe_3_O_4_@UiO-66-NH_2_ catalyst structure. Additionally, experiments with various blends of restaurant waste oil biodiesel and diesel at different torque levels in a CI engine demonstrated improved performance metrics (BTE, BSFC, and EGT) and reduced harmful emissions. The results indicated that blends of restaurant waste oil biodiesel and diesel favorably impact diesel engine applications. Dubey et al.^28^ employed RSM techniques to optimize diesel engine performance (BSFC, BTE) and emissions (smoke, NOx, CO, HC) using a waste-cooking soybean oil biodiesel-diesel blend with EGR variations. The optimized variables demonstrated enhanced performance and reduced emissions for all load settings. Similarly, the RSM optimal blend of 10% diethyl ether-90% B35-diesel blends with 15% EGR on stationary diesel engines showed improved BSFC (0.272 kg/kWh) and BTE (31.47%). Also, the reduced emission levels of smoke (18.94 HSU%), NOx (91 ppm), CO (0.03%), and HC (24 ppm), respectively^29^.

Based on previous literature, only a limited number of research papers have been published on the synthesis of biochar from various crops and its use as a catalyst for biodiesel production with suitable applications. It was established that no experiments had yet been conducted on using a GA to optimize a regression equation with a single target derived from RSM for producing PJBC using *Prosopis Juliflora* biomass waste to achieve a higher yield rate. Also, the PJBC has never been utilized as a green catalyst for biodiesel synthesis from any biomass waste feedstock. In order to fill the aforementioned research void, this study looked at the usage of GA to optimize the input variables and new PJBC catalysts to improve the trans-esterification method’s ability to produce biodiesel from waste *Trichosanthes cucumerina* seed bio-oil. Initially, the central composite design model in RSM is used to construct the pyrolysis run order for PJBC production. The PJBC yield regression equation is employed as an optimization function, and GA is applied to determine the optimal input parameters of pyrolysis. Numerous characterization investigations have verified and confirmed the catalytic activity of PJBC, including scanning electron microscope (SEM), FTIR spectroscopy, X-ray diffraction (XRD), Energy-dispersive X-ray (EDX) spectroscopy, Brunauer-Emmett-Teller (BET) method, XPS, and physico-chemical characteristics, respectively. Finally, the PJBC catalyst with high-performance trans-esterification makes efficient WTSB production, and the physico-chemical properties were evaluated to ascertain WTSB suitability as an alternative to current diesel fuels. Subsequently, the operating characteristics of a DI diesel engine were tested using diesel fuel and various WTSB/diesel fuel blends. The outcomes were then contrasted against existing diesel fuel results for various engine load settings. Also, the WTSB and their blends of environmental impact and lifecycle assessment were studied.

## Materials and methods

### Overview of *Prosopis juliflora* feedstock, PJBC production, and optimization

This section discusses the availability and selection of feedstocks, the preparation of PJBC, the prediction and optimization of PJBC synthesis using RSM-CCD and GA, and the evaluation of its physico-chemical characteristics.

#### Availability and specific reasons for selecting *Prosopis juliflora* feedstocks

**Fig. 2:**
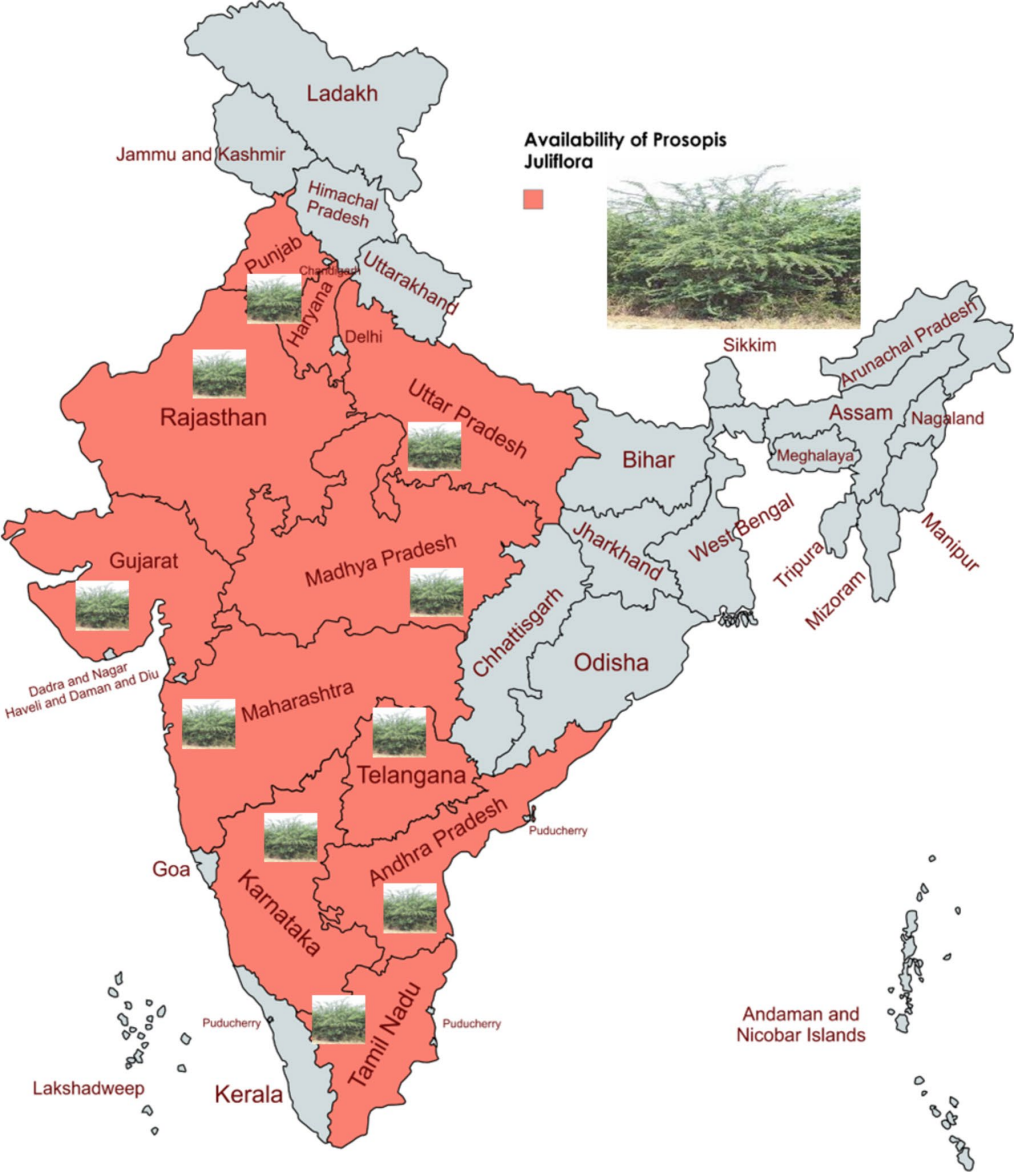
Availability of *Prosopis Juliflora* plant in India (map generated through https://www.mapchart.net/india.html).

In several Indian states, *Prosopis juliflora* biomass is publicly accessible, as shown in Fig. 2. A large portion of the biomass grown in this area comes from abandoned land and agricultural trash. It generates 35 to 45 tonnes of 5 to 6 years of species biomass per hectare. *Prosopis Juliflora* can tolerate high levels of pH, but beyond pH 9.0, the increasing alkaline content significantly reduced the germination rate of *Prosopis Juliflora* seed. Usually, 30–35 ^o^C is ideal for *Prosopis Juliflora* seed germination. Interestingly, germination quickly declines at temperatures less than 20 ^o^C or over 40 ^o^C^30^. The extensive growth of *Prosopis Juliflora* plants is available as waste biomass around the local village areas. This plant exhibits characteristics of a phreatophyte, mesophyte, or xerophyte depending on the water conditions in the area. *Prosopis Juliflora* has two root systems to take advantage of occasional rainfall: a deep taproot and a lateral surface root^31^.

The *Prosopis Juliflora* was selected as the biomass source for biochar production due to its abundance and accessibility, particularly from abandoned lands and agricultural waste in several Indian states (as shown in Fig. 2), ensuring a consistent and cost-effective supply of raw materials. Its biomass has a high caloric value (18.2 MJ/kg) and substantial lignin and carbon content, resulting in high yield and efficiency for biochar production, coupled with low moisture and ash content^9,32^. Utilizing *Prosopis Juliflora* also offers environmental benefits by managing this invasive species and converting it into a valuable resource. The biochar derived from *Prosopis Juliflora* exhibits excellent physico-chemical properties, with functional groups such as hydroxyl, carboxyl, and graphitic sp^2^ carbon enhancing its catalytic activity^33^. It has demonstrated the effectiveness of PJBC as a catalyst in biodiesel production, achieving high conversion rates and fuel properties that meet ASTM standards.

#### Preparation of PJBC

**Fig. 3:**
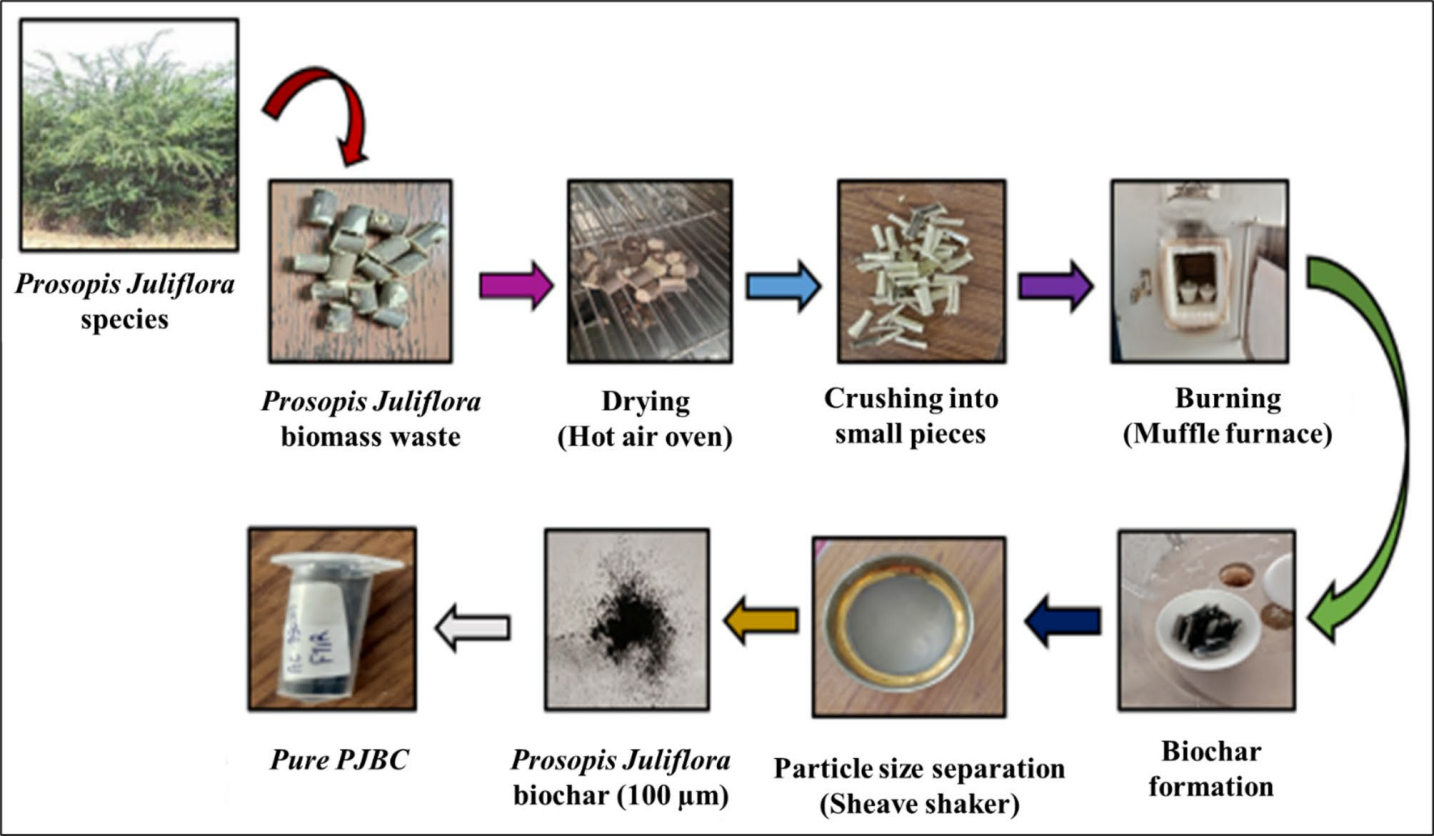
PJBC synthesis process layout.

For preparing the biochar, the wood samples of *Prosopis Juliflora* were collected from the adjacent side lands of agricultural cultivating areas. The stems of the gathered bushes were cut into evenly sized pieces and thoroughly cleaned with hot water to remove any dirt. Approximately throughout 3–4 days, the wood pellets are exposed to sunlight to naturally dry. After the natural drying, the collected wood pellets were kept in a forced air-circulating hot oven for regular drying and heated at 100 ^o^C for around 12 h. Overnight, the moistured-out stem portions are kept to cool down at atmospheric temperature. Later, the wood pellets are kept in a muffle furnace with a silica crucible led (50 mg) and heated to a carbonization temperature of 775 °C for an hour. As a result, the generated carbon was brought back to ambient temperature, crushed, and evenly sieved to 100 μm using a sheave shaker. Finally, the *Prosopis Juliflora* biochar was stored in a tightly closed vial to avoid the atmospheric reaction. The maximum yield of PJBC obtained by using the pyrolysis process is 48.93%. The complete process of synthesizing PJBC from *Prosopis Juliflora* biomass waste is presented in Fig. 3.

#### Response surface methodology (RSM)

The statistical methodology seeks to determine the optimal combination of input elements to improve any number of outcome variables. This entails maximizing processing variables within predetermined bounds for the best final results^26^. In regression analysis, the least squares approach is used to estimate the relationship between input elements and outcome variables, employing either a linear or quadratic polynomial, as shown in Eq. (1).

1 $$\:{Z=x}_{0}+\sum\:_{c=1}^{n}{x}_{c}*{a}_{c}+\:\sum\:_{c=1}^{n}\sum\:_{c\ge\:d}^{n}{x}_{cd}*{a}_{c\:}*{a}_{d}+C$$

Where Z = quadratic coefficient for result forecast; $\:{x}_{o}$ = value of bias; $\:{x}_{i}$ & $\:{x}_{ij}$ = linear and interactive coefficients; n = number of factors; $\:{a}_{c}$ & $\:{a}_{d}$ = independent variables; C = random error.

Based on the previous literature^10,15,34^, the ranges of process parameters involved in PJBC synthesis in the pyrolysis process were selected as 25 to 75 mg of *Prosopis Juliflora* wood weight, 750^o^C to 800 °C of carbonization temperature, and 30 to 90 min of process duration. Second-degree polynomial equations are frequently utilized to specify connections between elements and outcomes and simplify procedures. This process is facilitated through statistical and mathematical techniques provided by RSM. Design Expert 13.0.15 software was used to conduct the experiments, accurately recording responses and analyzing the results from a set of 20 different experiments designed with the CCD method. Table 2 details the experimental conditions, employing the CCD model to illustrate the three input factors at five distinct levels (-α for axial point, -1 for low, 0 for mid, + 1 for high, and + α for axial end).

**Table 2:** PJBC inputs and their levels.

PJBC inputs	Symbol	Levels				
		−α	−1	0	+ 1	+α
Weight (mg)	W	12.5	25	50	75	87.5
Temperature (°C)	T	737.5	750	775	800	812.5
time (min)	t	15	30	60	90	105

#### CCD modeling and its key analysis

A CCD matrix was developed to optimize PJBC production using the three input elements (W, T, and t) itemized in Table 2. This test matrix generated 20 runs, every block having six center points, as detailed in Table 3. The PJBC yield outcomes were derived from the experimental results by integrating the 3 input factors (W, T, and t) to establish each run order. To minimize random errors, each pair of PJBC inputs was replicated three times, and the CCD was fed with the average findings. After inputting the given data, regression analysis was performed to fit the data to the chosen CCD model. The resulting regression equation can be used to predict the outcome variable based on any arrangement of distinct variables within the design area, aiding in the determination of the ideal circumstances for PJBC production. Analysis of variance, regression assessment, and graphing of PJBC output are the three stages used by the CCD model to finish the prior operations. The residuals obtained by comparing the experimental PJBC yield with the predicted values are displayed in Table 3.

**Table 3:** CCD experimental test matrix for predicting PJBC yield.

Standard order	Run order	W: Weight (mg)	T: Temperature (°C)	t: time (min)	PJBC yield (%)		
					Experimental value	Predicted value	Residue
18	1	50	775	60	48.93	47.8	1.13
8	2	75	800	90	35.4	36.52	-1.12
9	3	12.5	775	60	26.21	25.13	1.08
3	4	25	800	30	36.1	38.12	-2.02
7	5	25	800	90	34.23	33.87	0.36
2	6	75	750	30	25.92	23.5	2.42
13	7	50	775	15	25.4	26.13	-0.73
10	8	87.5	775	60	23.54	24.47	-0.93
19	9	50	775	60	48.02	47.63	0.39
4	10	75	800	30	46.31	45.85	0.46
14	11	50	775	105	37.46	38.58	-1.12
5	12	25	750	90	22.15	23.11	-0.96
12	13	50	812.5	60	35.23	36.5	-1.27
16	14	50	775	60	47.59	47.01	0.58
1	15	25	750	30	23.24	23.89	-0.65
11	16	50	737.5	60	39.67	40.71	-1.04
17	17	50	775	60	48.12	47.5	0.62
15	18	50	775	60	47.43	47.91	-0.48
20	19	50	775	60	46.93	46.15	0.78
6	20	75	750	90	26.48	25.14	1.34

#### Genetic algorithm (GA)

Genetic algorithms (GAs) work on the principle of “survival of the fittest,” inspired by Darwin’s theory of evolution. It is an optimization technique based on search and genomics that uses a selection-based methodology. GAs use pairs of encoded variables to steer the optimization process rather than utilizing parameters directly. Using an objective function, the GA originally assessed each member of the population’s fitness. A tournament procedure was then employed to choose the best genes for the pairing pool. In this selection procedure, individuals were randomly picked to compete, and the parent who scored the highest on fitness was declared the winner. If more parents are needed, the process of choosing and adding them to the pairing pool is repeated. Variation in the breeding population is introduced through simulated binary crossover (SBX) and polynomial mutation^35^. New children are created by combining two well-chosen parents. The genetic exchange must account for both crossover and mutation, with a focus on achieving more crossover trials and fewer mutations. The GA model used in this study was configured with specific parameters to optimize the pyrolysis process. The algorithm was set to run for 60 generations with a population count of 100 individuals. A crossover index points of 10 was selected based on a probability value of 0.8 for crossover operations. The mutation process was also controlled using a 10-mutation index and a probability value of 0.2 for mutation trials.

**Fig. 4:**
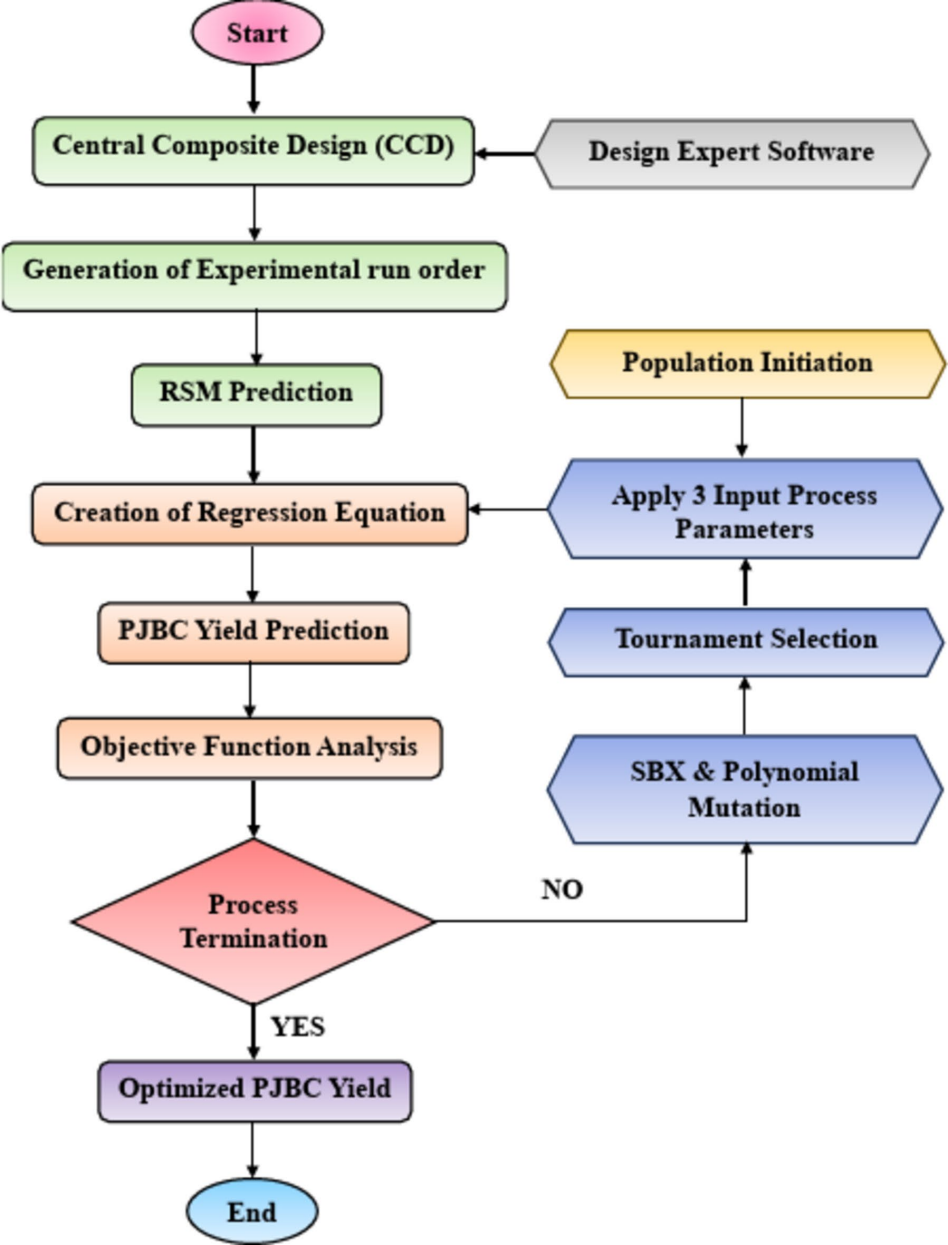
Workflow of the RSM Model Integrated with GA.

The distribution of probabilities ensures that the number of children produced by the parents is evenly divided, with an advanced likelihood that the kids will look a lot like the initial parent-provided answers. The previously described steps are repeated until the GA concludes. The process terminates upon the fulfillment of halting criteria, like finding the best answer or reaching a record number of generations. Utilizing MATLAB edition R2024a, the whole GA structure was created, and its flowchart is depicted in Fig. 4.

#### Physico-chemical characteristics of PJBC

Activated carbons are well-known for their ability to adsorb harmful substances in soil. The adsorption capacity of activated carbons is influenced by various physico-chemical properties such as pH, solubility, bulk density, M_c_, V_c_, A_c_, and FC_c_ content. The PJBC’s pH value was mostly alkaline, measuring 7.6, and its conductivity value was 436 µS/cm. This pH value is acceptable in various applications, including catalytic activity, water treatment, soil conditioning, and sugar decolourization. The bulk density value of PJBC was 0.45 g/mL, indicating that the substance had many pores. The effectiveness of adsorption increased as the number of holes increased, and more segments of porosity development reduced bulk density^36^. PJBC had a low % moisture content of 5.8%, indicating high adsorption capacity. The capacity of activated carbon to absorb moisture rises when it contains less water. The activated carbon with lower ash content is considered good quality. PJBC had a shallow ash content of 2.35%, indicating its good quality and enhanced adsorption capacity. Reduced mechanical strength and potential changes to the carbon’s ability to absorb water due to high ash concentration. PJBC had a volatile matter of 26.68% and a higher fixed carbon content of 65.17%. PJBC has poor solubility in both water and acid (15.24% and 11.65%, respectively). All the above characteristics of PJBC are expected to help catalytic activity, soil contamination and wastewater treatment involving a more significant pollutants’ adsorption.

### Feasibility evaluation of PJBC as a catalyst for WTSB production

The feasibility of employing sustainable synthesized biochar derived from *Prosopis Juliflora* biomass as a heterogeneous catalyst for biodiesel production was evaluated in this section by means of the trans-esterification method, fuel property evaluation, and comparison with existing diesel fuel for use in DI diesel engine applications.

#### Production and diverse applications of *Trichosanthes cucumerina* crop

The *Trichosanthes cucumerina* crop, sometimes referred to as snake tomato and viper gourd, is a member of the Cucurbitaceae family. The Latin term *Trichosanthes cucumerina L.* is where the expression “snake gourd” originates. It is extensively grown in Australia and Nigeria, as well as in a number of Asian nations, especially the Republic of Bangladesh, India, Nepal, China, and the island of Sri Lanka^37^. According to the Indian Horticulture Database for 2020-21, the production rate of snake gourd in India was approximately 16.18 tonnes per hectare. This extensive cultivation led to a significant amount of mature or waste *Trichosanthes cucumerina* crops being generated in horticulture fields and green markets. Snake gourd is a widely cultivated vegetable in India, with major growing regions including Maharashtra (Nashik, Pune, Ahmednagar), Tamil Nadu (Coimbatore, Tiruchirappalli, Madurai), Andhra Pradesh (Guntur, Krishna, Prakasam), Karnataka (Mysore, Tumkur, Bangalore), Kerala (Thrissur, Malappuram, Kottayam), West Bengal (Purba Medinipur, Howrah, Hooghly), Odisha (Cuttack, Balasore, Puri), and Uttar Pradesh (Gorakhpur, Basti, Azamgarh). This data is sourced from the Ministry of Agriculture and Farmers’ Welfare.

**Fig. 5:**
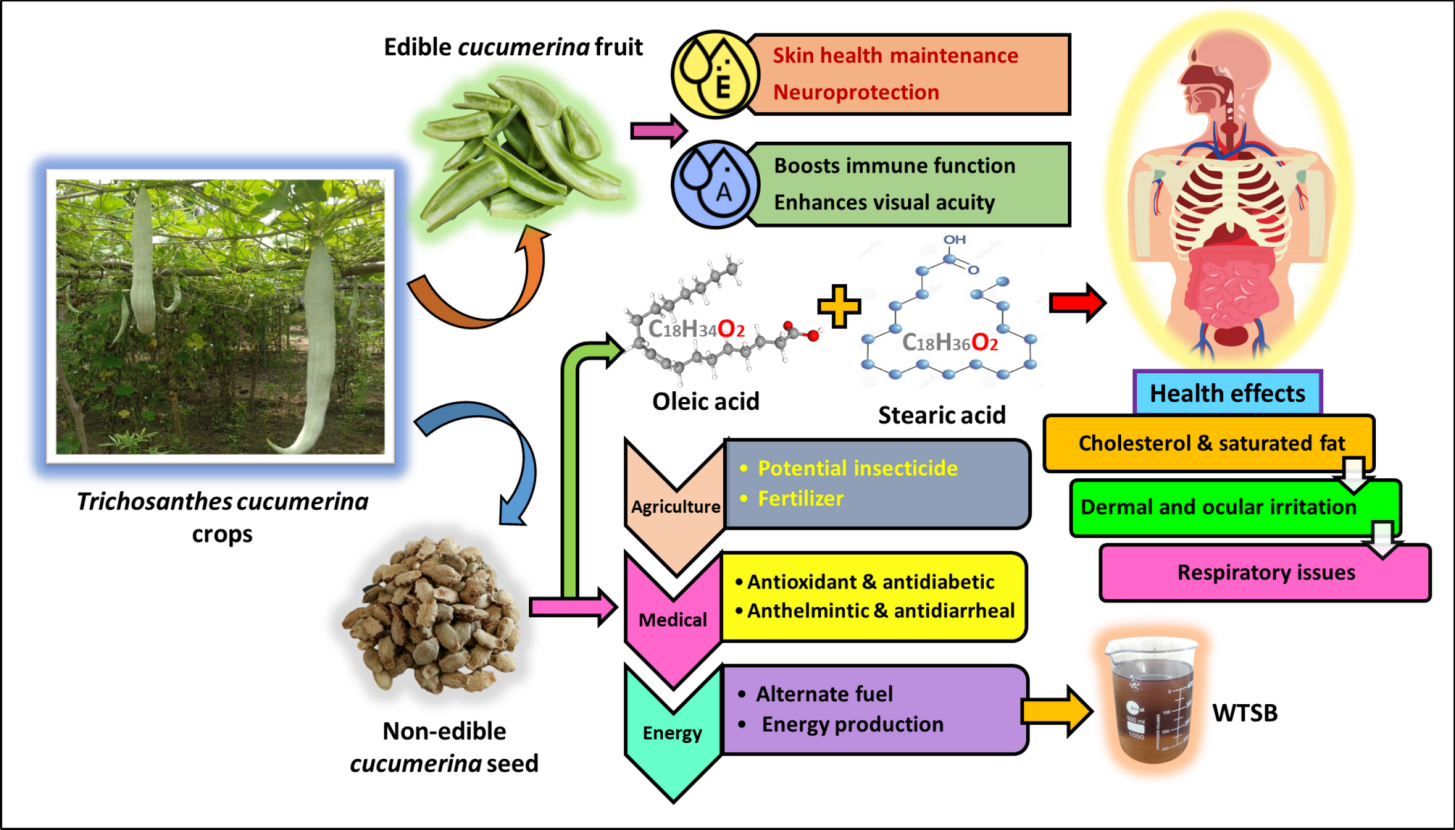
Various potential applications of *Trichosanthes cucumerina* crops.

A single *Trichosanthes cucumerina* fruit measures 40 to 120 cm in length, is pale green in color, weighs between 0.5 and 1 kg, and contains 40 to 70 seeds. They are valued for their nutritional content and are often utilized as a vegetable that may be eaten. The fruit has high levels of vitamins A and E, which contribute to improved immune function, better vision, preservation of brain tissue, and skin health^38^. However, waste *Trichosanthes cucumerina* seeds are generally not preferred for cooking or consumption. The seeds contain 52.73% unsaturated fatty acids, with a significant proportion of oleic acid (C_18_H_34_O_2_ = 41.27%) and stearic acid (C_18_H_36_O_2_ = 12.81%). These acids can negatively impact human health, potentially causing cholesterol issues, respiratory problems, and eye irritation, rendering the seeds non-edible. However, these inedible seeds are valuable for energy generation, medicine, and agriculture. The edible and non-edible products of *Trichosanthes cucumerina* crops, along with their valuable applications, are illustrated in Fig. 5. Building on the diverse applications of *Trichosanthes cucumerina*, this research focuses on the production of biodiesel from its waste seeds.

#### Extraction of waste *Trichosanthes cucumerina* seed bio-oil (WTSO)

**Fig. 6:**
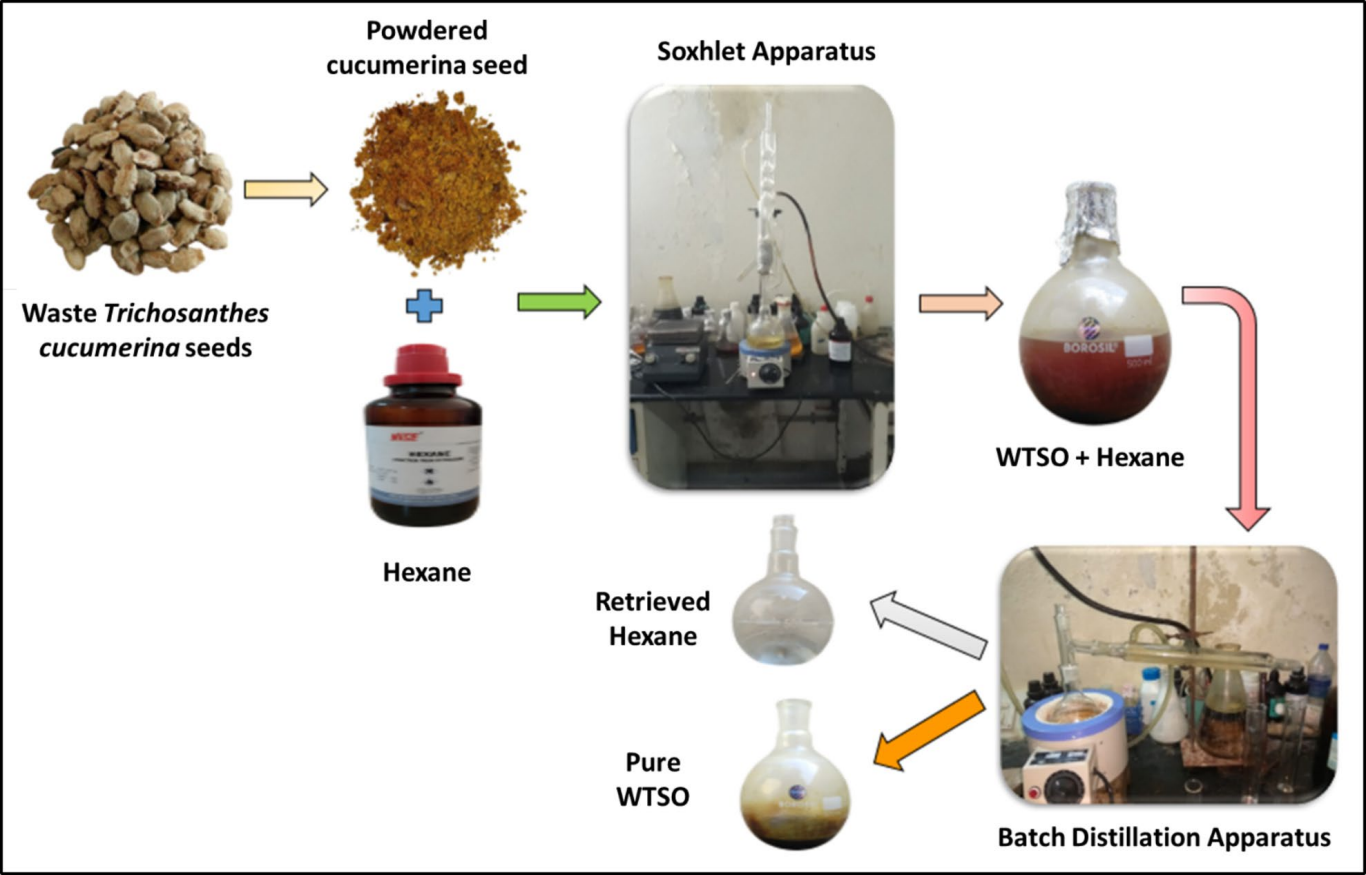
Pure WTSO extraction process layout.

*Trichosanthes cucumerina* waste seeds are gathered from gardening fields and organic markets and used to make the WTSO. After separating the waste or useless seeds from the kernel fruit is washed, dehydrated, and coarsely powdered. Using a Soxhlet apparatus made of borosilicate glass, 200 g of pulverized seeds are placed in a thimble, and hexane (280 mL) is used to extract WTSO. An apparatus of Dimroth reflux circulates cooling water to condense solvent vapors, which are then returned to the chamber. Hexane is evaporated by heating the WTSO-hexane combination to 68 °C, leaving pure WTSO in the distilling flask. Approximately 210 mL of hexane is recovered for reuse. Figure 6 illustrates a mean of 28.43% (WTSO) obtained from three extraction cycles and the properties detailed in Table 4.

**Table 4:** Pure WTSO properties.

Fuel properties	WTSO	Instrument used	ASTM standard
Molecular formula	C_41_H_82_O_12_	GC-MS analyzer	-
Molecular weight (g/mol)	814.38		
Kinematic viscosity (mm^2^/s)	42.85	Redwood viscometer	D445
Density (kg/m^3^)	912	Density measurement	D1298
Heating value (MJ/kg)	40.36	Bomb calorimeter	D240
Flashpoint (°C)	236	Closed cup Pensky Martens apparatus	D93
Fire point (°C)	262		
Cetane number	45	Traditional laboratory method	D976
Acid value (mg KOH/g)	5.16	Colorimetric titration	D664

#### Trans-esterification method for WTSB production and its fuel properties evaluation

**Fig. 7:**
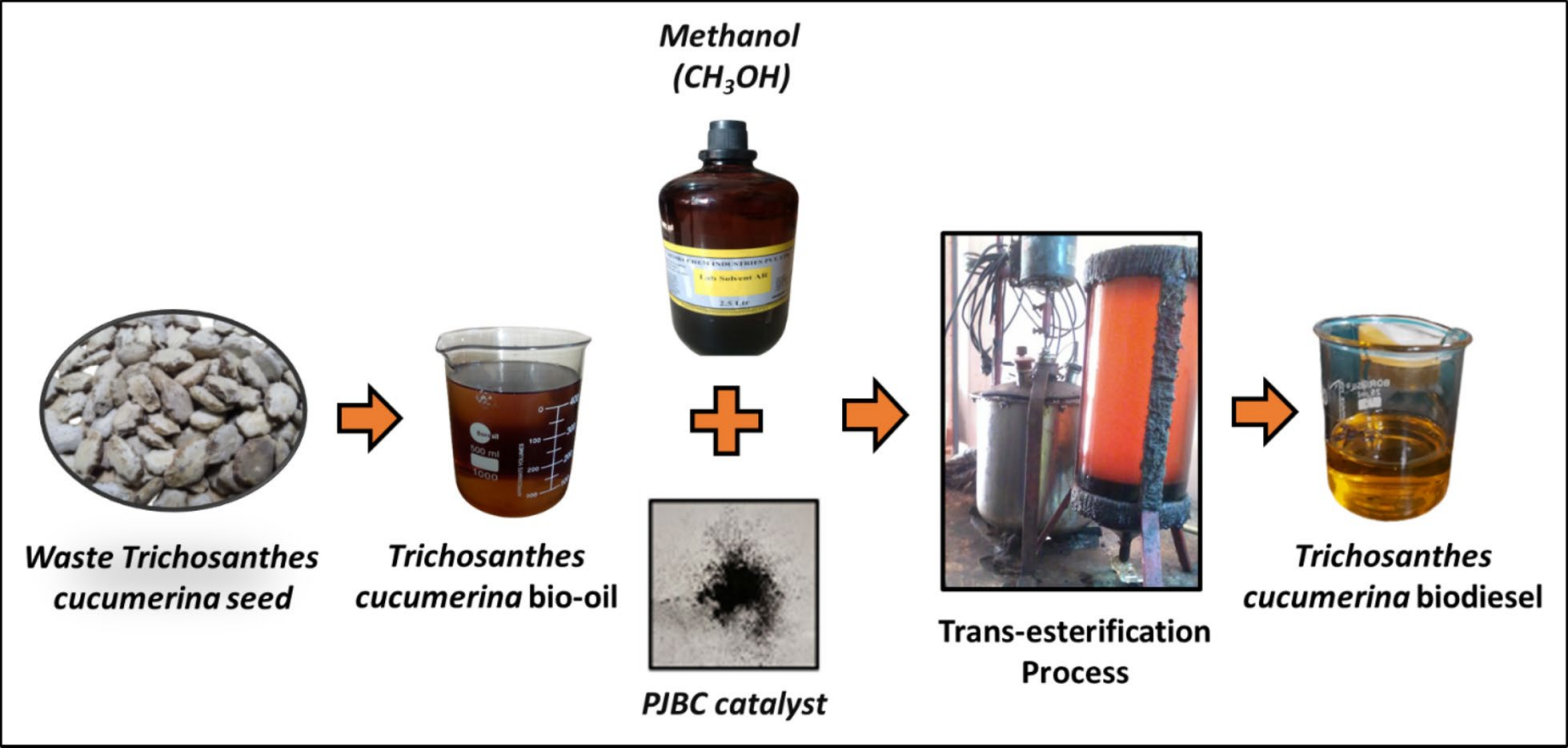
Process of producing WTSB.

The bio-oil is produced from waste and nonedible seeds of *Trichosanthes cucumerina* fruit through the Soxhlet extraction method. An extracted waste *Trichosanthes cucumerina* seed bio-oil (WTSO) is subjected to transesterification, and their step-by-step process is presented in Fig. 7. In this process, the 600 g CH_3_OH and 1000 g WTSO were taken with 13:1 methanol to WTSO molar proportions. At the initial stage, the 4 wt% of PJBC catalyst and 600 g of CH_3_OH were kept in the Erlenmeyer flask with a stir bar and were placed on a stirring hot plate to mix until the PJBC catalyst was completely dissolved in the methanol. After that, the 1000 g WTSO was taken in another Erlenmeyer flask, which was held on the hot plate and heated. Once the WTSO reached 60 °C temperature, the methanol/PJBC catalyst mixture was added drop by drop into the heated WTSO. This mixture was completely stirred for the next 60 min at a moderate speed by holding the heating temperature at 60 ^o^C. Then, the mixture was transferred into a separatory funnel, allowing it to cool down and phase separation for 20 min. The lower layer of glycerol is removed from the bottom of the separatory funnel, and the upper layer of waste *Trichosanthes cucumerina* seed biodiesel (WTSB) is collected in the Erlenmeyer flask. Finally, the trace percentage of glycerol with some catalyst present in WTSB is removed through purification, which produces pure WTSB with a maximum yield of 97.42%.

The physico-chemical fuel properties of WTSB are measured as per ASTM standards and compared with existing diesel fuel properties, which are tabulated in Table 5. It shows that the produced WTSB and its blended fuels exhibit several physico-chemical characteristics similar to diesel fuel already in use. Notably, all the WTSB fuel properties are within the ASTM D6751 limit for biodiesel characteristics. It was supported that the density of WTSB is very close to existing diesel fuel, enabling more efficient fuel atomization and faster A/F mixing^39^. Being well within ASTM tolerances, the kinematic viscosity (3.86 mm^2^/s) guarantees the proper fluidity and prevents problems such as the development of bigger droplets during fuel injection and atomization^40^. WTSB fuel exhibits 52% and 45% greater flash and fire points than current diesel fuel, indicating its safety for transportation and storage in low-temperature environments. The WTSB reaches more than 95% of the heating value of the diesel fuel, indicating a significant energy content. It may improve the fuel combustion characteristics and lower energy usage for a cycle of engine operation. The high cetane number (54) of WTSB ensures a higher ignition quality than diesel. Furthermore, the WTSB blends also have fuel properties similar to those of existing diesel fuel.

**Table 5:** Comparison of WTSB physico-chemical fuel properties with existing diesel fuel.

Fuel characteristics	ASTM D6751	WTSB100	WTSB75	WTSB50	WTSB25	Diesel	Analysis method	ASTM norms
Kinematic viscosity (mm^2^/s)	1.9–6.0	3.86	3.52	3.27	3.04	2.71	Redwood viscometer	D445
Density (kg/m^3^)	860–900	851	849	844	841	836	Density measurement	D1298
Heating value (MJ/kg)	–	39.12	40.73	41.61	42.19	42.50	Bomb calorimeter	D240
Flash point (°C)	130 min	143	127	108	75	68	Closed cup Pensky Martens apparatus	D93
Fire point (°C)	–	157	148	127	98	86		
Cetane number	47 min	54	52	51	49	48	Traditional laboratory method	D976
Acid value (mg KOH/g)	0.5 max	0.42	0.26	0.21	0.13	0.05	Colorimetric titration	D664
Ultimate analysis								
Carbon (C) level (%)		70.59	75.17	77.5	80.2	83.3	CHNS (O) analyzer	D5291
Hydrogen (H) level (%)		13.72	14.3	14.6	14.8	15.3		
Nitrogen (N) level (%)		–	–	–	–	–		
Sulfur (S) level (%)		–	–	–	–	0.01		
Oxygen (O) level (%)		15.71	8.1	7.7	4.11	–		
Calculated C/H ratio		5.14	5.25	5.31	5.38	5.44		

The results of the ultimate analysis from WTSB, WTSB blends and existing diesel fuel are reported in Table 5. It was done by CHNS (O) analyzer is useful for calculating the proportions of Carbon (C), Hydrogen (H), Nitrogen (N), Sulfur (S), and Oxygen (O) in organic compounds. It works on the basis of the “Dumas method,” which entails completely oxidizing the sample instantly by “flash combustion”. The various elements in WTSB were analyzed and compared with current diesel fuel, resulting in a 12.81% lower carbon level in WTSB than existing diesel fuel. It might be the disparity ascribed to changes in feedstock sources and fuel production methods. The higher percentage of oxygen level (15.71%) in WTSB has been ensured through the formation of most ester groups in its chemical composition. The abundance of oxygen in WTSB enhances all aspects of combustion, including fuel atomization, vaporization, and A/F mixing. In addition, a high percentage of hydrogen (15.30%) was found in existing diesel fuel, reducing the C/H ratio by 5.14 in WTSB. The WTSB’s decreased C/H ratio and plentiful supply of atmospheric oxygen components improve DI combustion and oxidization of fuel in the energetic pockets, which lowers smoke emissions^41^. The WTSB could not identify any levels of sulfur or nitrogen content, indicating that no sulfur or nitric oxide emissions were produced during the combustion of WTSB^42^. These findings underscore WTSB’s adherence to industry standards, supporting its suitability as a biodiesel fuel and could be used as a replacement for existing diesel fuel.

#### Challenges in scaling the PJBC-based process for commercial WTSB production

The study highlights the high yield and effectiveness of WTSB produced using PJBC catalysts, achieving a maximum WTSB yield of 97.42% with properties conforming to ASTM D6751 standards. This demonstrates WTSB’s potential as an alternative fuel source. However, scaling the PJBC-based process for commercial WTSB production poses challenges. Ensuring consistent recovery and reusability of the PJBC catalyst at scale is crucial to avoid cost increases and loss of catalytic efficiency. Additionally, sustainable sourcing of Prosopis Juliflora biomass must be managed carefully to prevent ecological imbalance or competition with other uses. The transition from laboratory-scale to large-scale production could also lead to increased costs due to additional processing requirements and quality control measures. Furthermore, while the WTSB blends exhibit favourable emission profiles, handling by-products like glycerol and adherence to environmental regulations add complexity to the process. Addressing these challenges through process optimization, technological advancements, and comprehensive life-cycle assessments will be essential for the successful commercialization of this WTSB production method.

## Experimental setup and operating procedure for DI diesel engine

An experimental analysis is conducted using a single-cylinder, water-cooled, four-stroke, direct injection (DI) diesel engine, which is paired with a Techno Mech eddy current dynamometer. The detailed technical specifications of the DI diesel engine are provided in Table 6.

**Table 6:** Technical specifications of DI diesel engine.

Manufacture and model of computerized engine	Kirloskar and TV1
Type of computerized engine	One-cylinder, four-stroke, computerized DI, water-cooled diesel engine
Cylinder diameter and stroke	B = 87.5 mm and S = 110 mm
DI engine’s compression ratio	17.5:1
DI engine power and running speed	5.2 kW and 1500 rpm
Cubic capacity of DI engine	661 cm^3^
Injector control pressure	200 bar
Injection timing	23° CA bTDC
Connecting rod and dynamometer arm length	*l* = 234 mm and R_m_ = 185 mm
Combustion chamber bowl design	Hemispherical
No. of injector holes and their diameter	*n* = 3 holes & d = 0.3 mm
Dynamometer make, model and type	Techno Mech, TMEC-10 and Eddy current dynamometer

The chosen computerized engine runs at 1500 rpm continuously under varying load conditions. Experimental tests were performed at five different engine load levels: 0%, 25%, 50%, 75%, and 100%. These loads were applied using an eddy current dynamometer and tested with both neat diesel and WTSB/diesel blends. The engine setup is equipped with an airflow indicator to gauge the rate of airflow and a fuel tank metering stack to gauge the amount of fuel used per unit of time. The computerized engine’s piston bowl is hemispherical in design, with cylinder overhead valves actuated by pushrods.

**Fig. 8:**
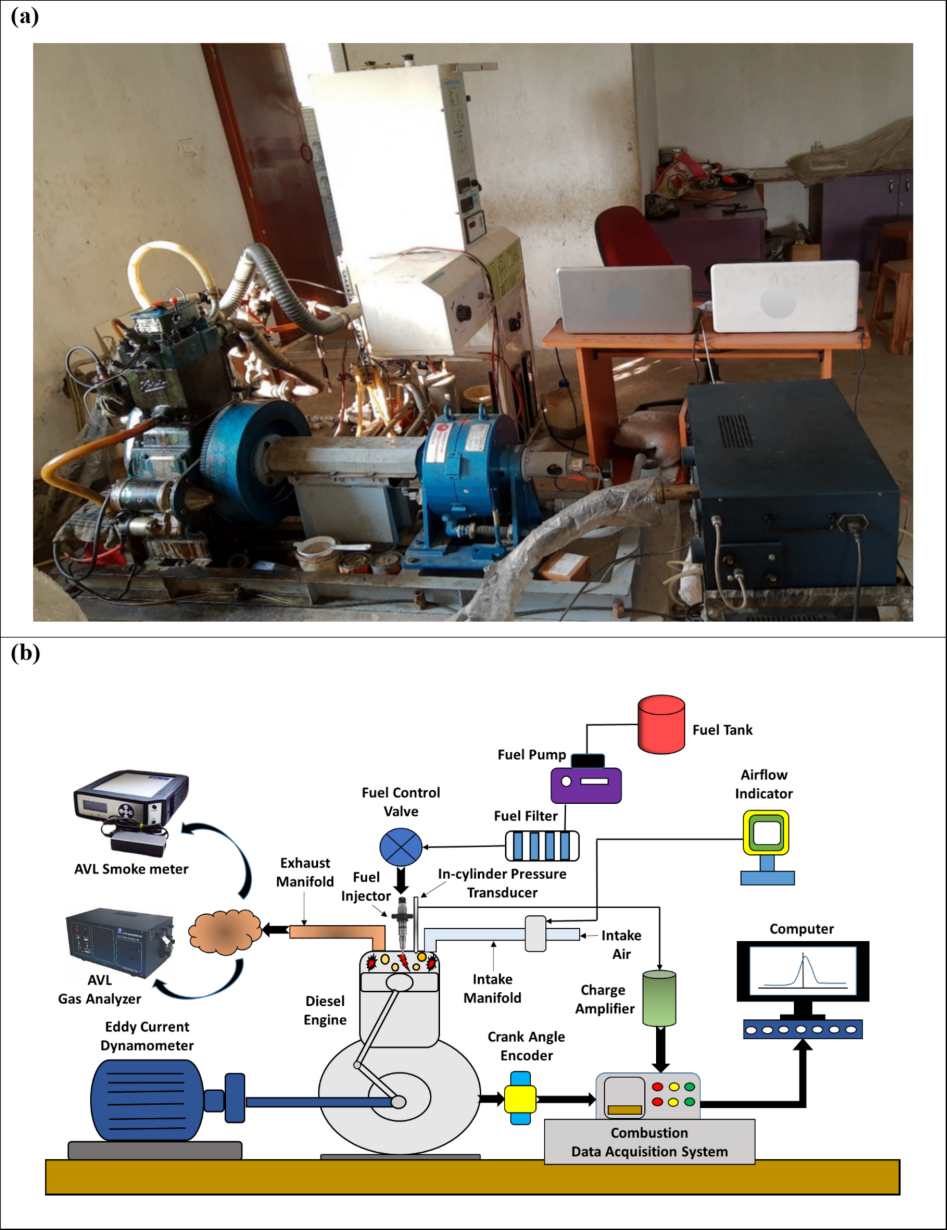
(**a**) Visual perspective and (**b**) Schematic architecture of DI diesel engine setup.

According to the engine manufacturer’s testimonial, fuel injection is set at 23° CA before TDC with a controlled injection of 200 bar. The DI diesel engine includes a crank angle encoder (AVL 364) and an in-cylinder pressure transducer (AVL GH12D). These instruments are connected to a combustion data acquisition system (DAQ), which is linked to a digital computer for recording combustion data. The “Engine Soft” software is used to obtain performance results of the DI diesel engine. Engine exhaust emissions, including S_HC_, S_CO_, and S_NO_, are measured using an AVL gas analyzer. Additionally, a smoke meter (AVL 437 C) is used to measure smoke opacity. The visual perspective and schematic architecture of the DI diesel engine experimental setup are illustrated in Fig. 8(a) and (b).

The DI diesel engine was tested at load conditions of 0%, 25%, 50%, 75%, and 100% while maintaining a constant speed of 1500 rpm. Prior to beginning the experiments, checks were performed on the DI diesel engine’s running temperature, coolant flow, lubricating oil flow, and test fuel (diesel and WTSB blend) levels. The loading accuracy of the Techno Mech eddy current dynamometer was verified and calibrated. The DI diesel engine is first run for 30 min to attain stable circumstances. Once the engine reaches stability, tests are performed using neat diesel and various WTSB blends across different load conditions. Each test is repeated three times per fuel blend to ensure accuracy, with the average values used for plotting graphs and verifying repeatability.

All measuring instruments utilized in this experimental analysis were regularly calibrated to ensure precision in the measurements. Analytical methods were employed to estimate the uncertainty arising from random or fixed errors during the experimental test. These errors can arise for several reasons, including but not limited to choosing equipment and inspection, human evaluation of data, and workplace conditions^43^. To verify the high accuracy of the measuring instruments, each experiment was performed three times with diesel, WTSB and its blends, and the average values were used for subsequent analysis. Percentage uncertainties were assessed for all testing parameters of engine load (L), speed (N), brake-specific fuel consumption (BSFC), brake thermal efficiency (BTE), S_HC_, S_CO_, S_NO_ emissions, and smoke opacity. Using Eq. (2), the total uncertainty of the experimental test is evaluated for all parameters based on the square root methodology^44^.

2 $$\begin{aligned} & =\sqrt{\begin{array}{c}{\left(\text{B}\text{P}\right)}^{2}+{\left(\text{N}\right)}^{2}+{\left(\text{B}\text{S}\text{F}\text{C}\right)}^{2}+{\left(\text{B}\text{T}\text{E}\right)}^{2}+{\left({\text{S}}_{HC}\right)}^{2}+{\left({\text{S}}_{CO}\right)}^{2}+{\left({S}_{NO}\right)}^{2}+{\left(\text{S}\text{m}\text{o}\text{k}\text{e}\:\text{o}\text{p}\text{a}\text{c}\text{i}\text{t}\text{y}\right)}^{2}\\\:.\end{array}} \\ &=\sqrt{{\left(0.1\right)}^{2}+{\left(0.5\right)}^{2}+{\left(2\right)}^{2}+{\left(2\right)}^{2}+{\left(1.5\right)}^{2}+{\left(0.7\right)}^{2}+{\left(1\right)}^{2}+{\left(1\right)}^{2}} \\ & = \pm 3.60 \% \end{aligned}$$

## Results and discussion

This section discusses the effects of process parameters on higher PJBC yield rate prediction and optimization, the various characterization studies on synthesized PJBC-based catalysts and their catalytic activity for biodiesel production, and their suitability for engineering applications.

### PJBC catalyst RSM-CCD prediction and GA optimization

In this section, RSM-CCD is used to predict the process parameters for PJBC catalyst synthesis, and GA optimization is employed to find their optimal values.

#### ANOVA and its effects on PJBC yield

Table 7 shows the ANOVA for PJBC yield with an inverse exponential model based on the prediction findings. The generated model had an F-value of 30.10, indicating strong significance. There is a 0.01% chance that noise will produce an F-value high. A p-value < 0.05 signifies the significance of the model. In this case, parameters like W, T, t, W×T, W×t, T×t, W^2^, T^2^, and t^2^ significantly impacted PJBC yield. In comparison to the pure error, the lack of fit’s F-value was non-significant at 2.63, making the CCD model highly robust with non-significant. The p-value < 0.05 and the lack of fit > 0.05 impacted the PJBC yield.

Moreover, if an experiment can be replicated well and the fit statistics’ R^2^ value is more than 0.9, the developed CCD model is said to have high correlations. According to the experiment’s ANOVA results and correlation coefficient value (R^2^), the constructed CCD model variation in output results is accurately categorized. The developed CCD model’s R^2^ score (0.9756), which is near to 1, indicates that it is deemed acceptable. It is possible to deduce from the ANOVA results that the combined effects of weight, temperature, and time on the *Prosopis Juliflora* biomass have a more significant effect on PJBC yield, as their p-values < 0.05. The yield estimation formula for PJBC, created by iterative statistical regression, is shown in Eq. (3).

3 $$\:\text{P}\text{J}\text{B}\text{C}\:\text{y}\text{i}\text{e}\text{l}\text{d}\:\left(\text{\%}\right)=47.16-0.052\text{W}+1.78\text{T}+0.768\text{t}-1.32\text{W}\times\:\text{T}-0.3\text{W}\times\:\text{t}-0.9\text{T}\times\:\text{t}-8.75{\text{W}}^{2}-4.73{T}^{2}-5.84{t}^{2}$$

**Table 7:** ANOVA for PJBC yield.

Source model	Sum of squares	df	Mean square	F-value	*p*-value
Model	7566.74	5	1513.35	30.10	< 0.0001 (significant)
W: Weight	2965.47	1	2965.47	58.98	0.0001
T: Temperature	2361.35	1	2361.35	46.97	0.0002
t: time	2134.28	1	2134.28	25.13	0.0016
W×T	968.45	1	968.45	19.26	0.0032
W×t	653.07	1	653.07	12.93	0.0451
T×t	842.72	1	842.72	81.52	0.0053
W^2^	1355.34	1	1355.34	26.96	0.0013
T^2^	893.10	1	893.10	17.76	0.0040
t^2^	1106.45	1	1106.45	8.96	0.0135
Residual	351.93	7	50.28		
Lack of fit	0.3508	3	0.1693	2.63	0.0751 (not significant)
Pure error	1.13	4	0.2827		
Cor total	7918.67	12			
R^2^ value	0.9756				
Adjusted R^2^ value	0.9438				
Predicted R^2^ value	0.8703				

**Fig. 9:**
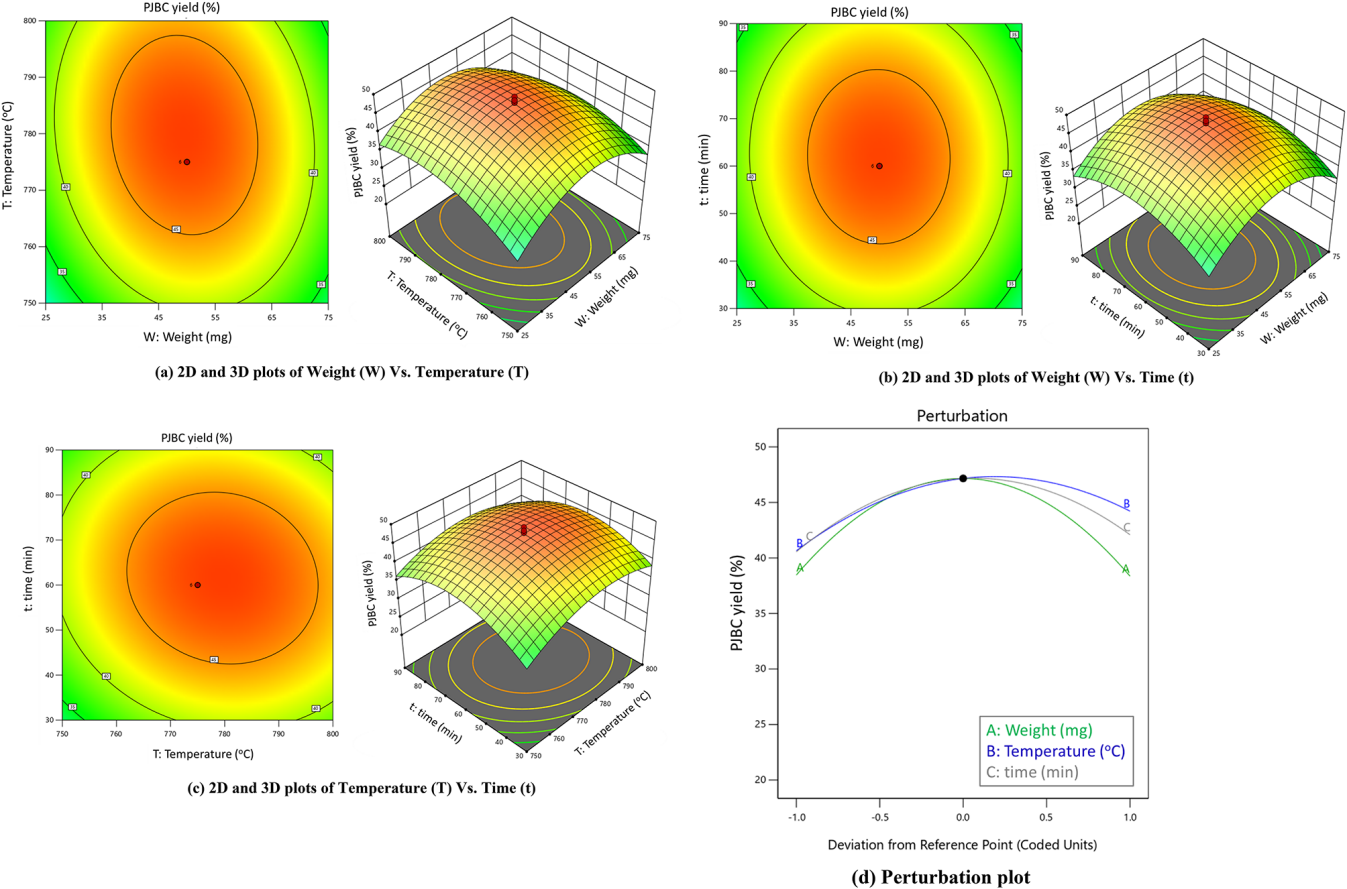
3D plots indicate the interaction effects of (**a**) Weight (W) Vs. Temperature (T); (**b**) Weight (W) Vs. Time (t); (**c**) Temperature (T) Vs. Time (t) and (**d**) perturbation graph on PJBC yield.

Based on the analysis of the 2D and 3D plots of Fig. 9 (a), it is clear that adjustments in Weight (W) and Temperature (T) with a fixed synthesis time of 60 min have a significant impact on the yield of PJBC. Specifically, increasing the weight from 25 to 50 mg and raising the temperature from 750 to 775 ^o^C can gradually increase biochar yield from 22.10 to 48.93%. Nevertheless, an extra 75 mg rise in weight would lead to a significant decrease in PJBC yield by 35.40%. This is because the carboxylic and hydroxyl chains aggregate to form bigger particles rather than separate forms, reducing the number of carboxyl group pairs and eventually reducing PJBC yield. Additionally, the increase in the value of PJBC extract leads to a 23.54% drop in biochar production. This is due to the possibility that the excess reducing agents in PJBC might result in the creation of smaller carboxyl groups or completely prevent the synthesis of PJBC.

The 2D and 3D plot findings in Fig. 9 (a) demonstrate how the PJBC yield rises gradually to a midlevel value of 50 mg. At this point, the maximum yield of 48.93% biochar is achieved. However, beyond this stage, the proportion of PJBC production drops to 46.31%. Hence, the mid-point action creates more PJBC yield than the minimum and maximum levels in weight and temperature. The quick heat transfer from the hot to the cold end is slowed down when the particle size rises because of the increased distance between the biomass’s surface and core. This temperature gradient favours the PJBC yield. Additionally, when the size of the particles increases, the vapour created by the thermal cracking of biomass must pass through the PJBC layer over a greater distance, which leads to more secondary reactions and the production of additional PJBC^45^.

Figure 9 (b) displays the relationship between Weight (W) and Time (t) at a constant burning temperature of 775 ^o^C. It is evident that lesser PJBC yields, 25.92% and 26.48%, are produced by the maximum and lowest biomass weight quantities, while higher PJBC yields (48.02%) are produced by the biomass weight intensity at the mid-value. The production of PJBC is progressively increased by lengthening the burning duration of *Prosopis Juliflora* biomass up to a certain point, beyond which PJBC yield falls gradually. A lower temperature associated with a high vapour residence time is necessary to maximize PJBC production. Allowing a longer vapour residence time facilitates the repolymerization of biomass constituents, as it provides ample time for the reactions to occur. Conversely, if the residence time is shorter, the repolymerization process remains incomplete, leading to a reduced PJBC yield^46^.

Figure 9 (c) shows the contact of Temperature (T) and Time (t) at a fixed *Prosopis Juliflora* biomass quantity of 50 mg, resulting in the yields of PJBC being enhanced by raising both parameters to their optimal values. The maximum PJBC yield at an optimal point is 48.12%. After that, from the optimal point, there is a slight decrement in the yield rate was observed from the 3D interaction plot. A high PJBC yield can only be achieved at medium temperatures, as high temperatures can induce the biomass to release its volatile components more energetically than the bond cessation energy. Less PJBC yield results from these volatiles escaping as gases^47^.

The influence of Weight (W), Temperature (T), and Time (t) on biochar yield is shown by the perturbation graph in Fig. 9 (d). The findings on the direction of progress towards reaching the maximal output response are supported and confirmed by the perturbation plot.

**Fig. 10:**
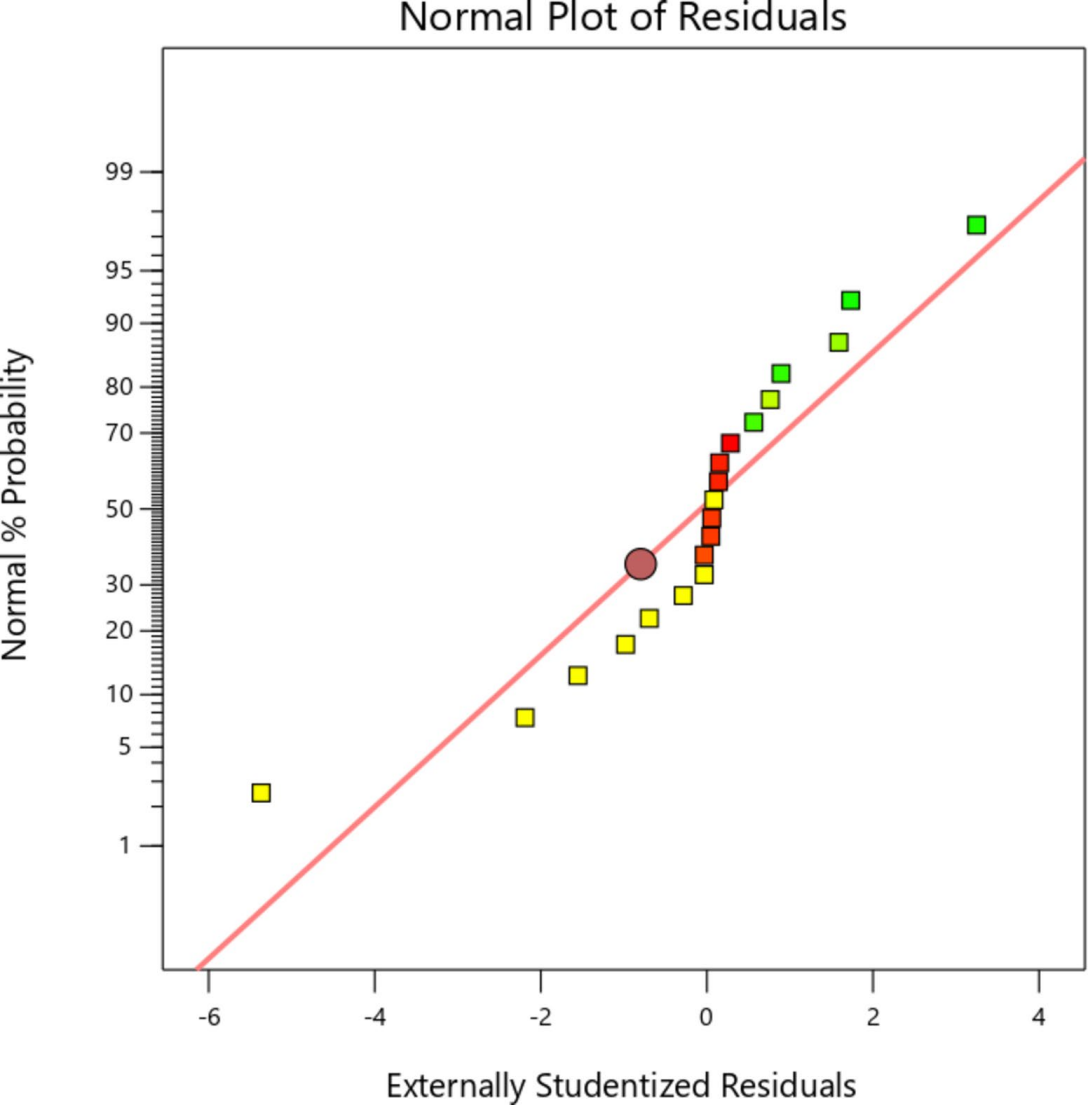
Normal plot of residuals for PJBC yield.

Figure 10 displays the graph between the PJBC yield in experimental outputs and predicted values. The graph shows that the data points of the predicted model value of PJBC yield, which match the observed experimental outcomes, are evenly distributed and appear to form a neat, straight line. This is likely due to the high level of agreement between the predicted R^2^ value (0.8703) and the adjusted R^2^ value (0.9438). Moreover, the signal-to-noise ratio of the developed CCD model is 26.715, which exceeds the target value of 4, indicating a satisfactory signal. Thus, the CCD model is suitable for exploring the design space.

#### Optimization of PJBC synthesis parameters through GA

**Fig. 11:**
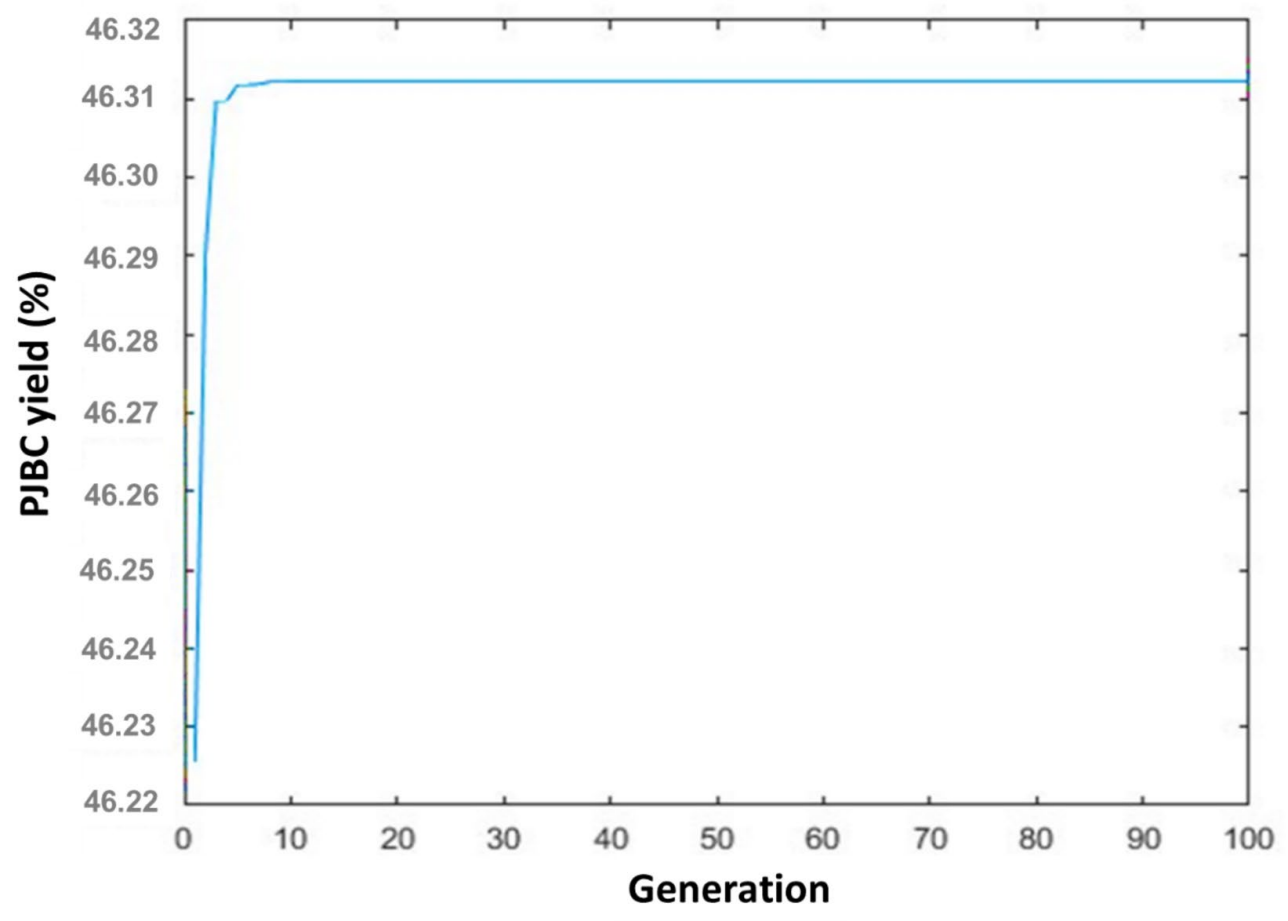
GA optimization plot for PJBC yield.

The PJBC yield iterative statistical regression Eq. (3) was used as the objective function of this optimization technique. It is used for the real-coded GA maximization constraint. Section 2.1.5 examined several elements of the GA model architecture as well as the methodology used in this optimization study. Figure 11 illustrates how the objective function converges to achieve PJBC yield as the quantity of generations expands. It reveals that the *Prosopis Juliflora* biomass achieves the optimal PJBC yield of 46.31% within the first ten generations. Hence, an adequate limit of 50 populations was utilized for optimization. Table 8 lists the most effective conditions for the highest PJBC formation during pyrolysis. Under optimized input elements of 60 mg / 790 °C / 60 min, the maximum PJBC yield of 46.31% was achieved in the pyrolysis process.

**Table 8:** GA optimized PJBC input variables.

PJBC input parameters	Unit	Symbol	Optimized input parameters
Weight	mg	W	60.1026 ≃ 60
Temperature	°C	T	791.0494 ≃ 790
Time	min	t	61.0031≃ 60

### Characterization studies on PJBC-based catalyst

The different characterization studies on PJBC-based catalysts were performed to ensure their fractures and grains, different chemical formations, crystalline size, inorganic groups, surface characteristics, and oxidation states were discussed in this segment.

#### SEM, FTIR, and XRD analysis of PJBC

**Fig. 12:**
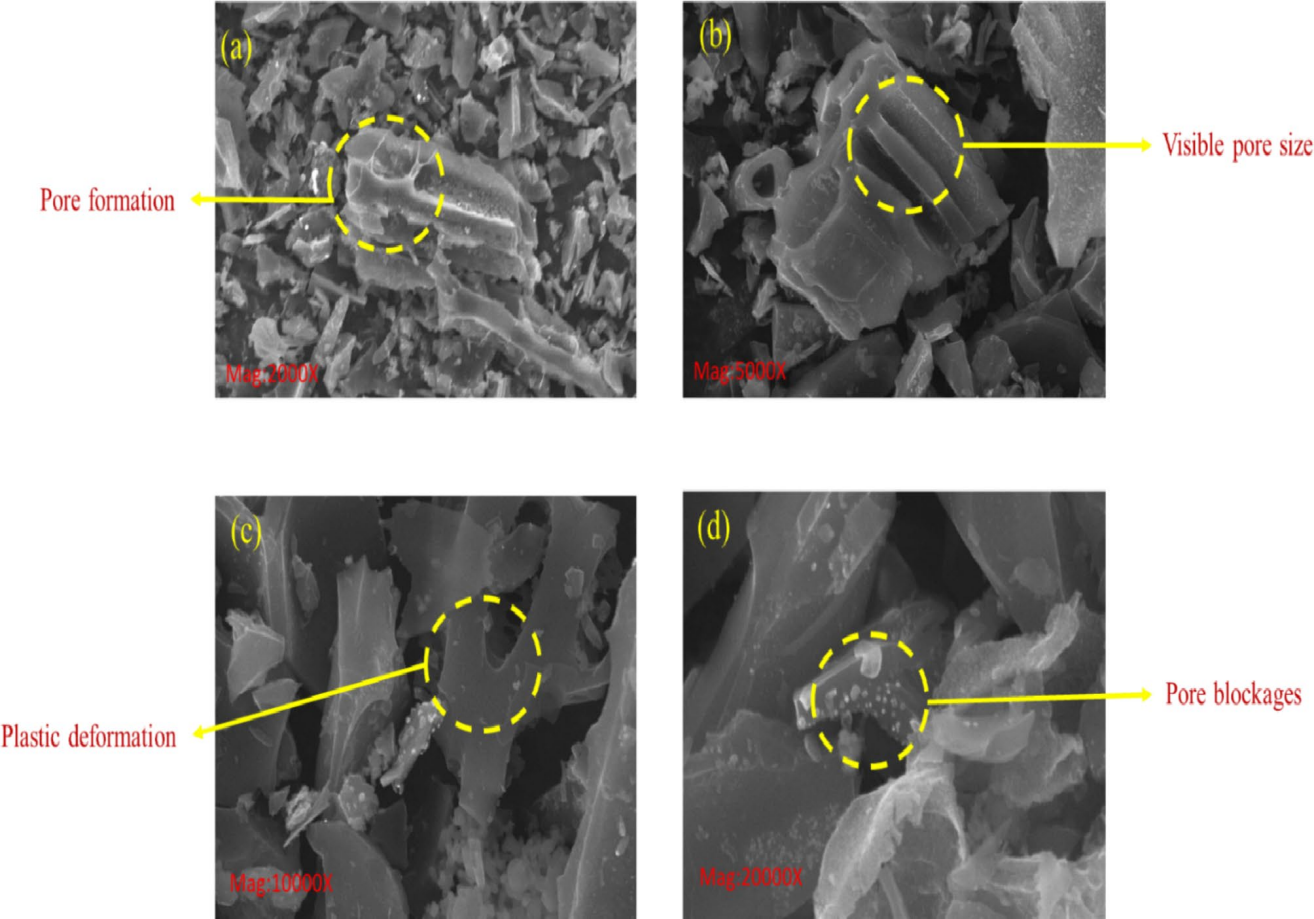
(**a**) SEM image of PJBC with ×2000, (**b**) ×5000, (**c**) ×10,000, and (**d**) ×20,000 magnifications.

Scanning electron microscopy (SEM) technology has a high-resolution capability, with image resolutions as low as 15 nm. The SEM results were captured at a width of 1 μm, using various magnifications including 2000X, 5000X, 10000X, and 20,000X, making CAREL ZEISS of model EVO18 for solids and thin films. This enabled the unambiguous identification of fractures and grains in the PJBC sample. SEM was used to analyze the PJBC sample, revealing porousness, substance, and surface characteristics. The images showed that PJBC has pores varying from 6.96 μm to 8.56 μm at 2000X, as shown in Fig. 12(a), which could be due to the exothermic process that occurred during the termination process, leading to pore formation. While in the 5000X magnification, as represented in Fig. 12(b), the observable pore diameters were also caused by the fast release of volatile materials during the pyrolysis process at extremely high temperatures. However, if PJBC is treated at higher temperatures owing to PJBC’s plastic nature with the view of 10000X magnification as shown in Fig. 12(c). From the 20000X magnification of Fig. 12(d), it may lead to pore blockages owing to the melting of the mineral’s forms PJBC. The availability of pores in PJBC makes it sound like a filter media for enhancing catalytic activity and soil quality studies^8^. Monisha et al.^48^ found a similar pore size on their biocatalyst and confirmed the increased catalytic reaction rates in biodiesel production.

**Fig. 13:**
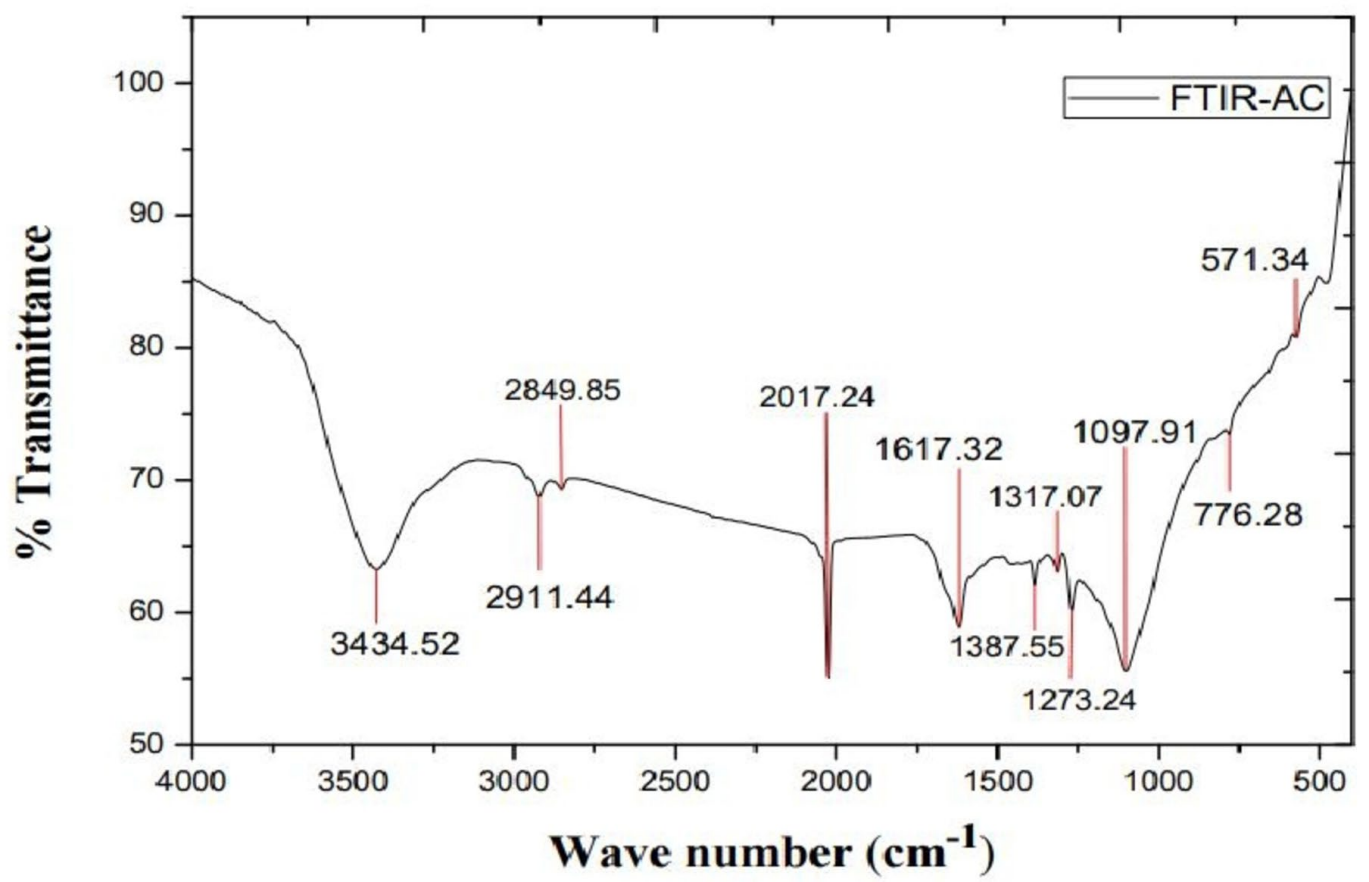
FTIR report of PJBC.

FTIR uses vibrations to discover organic compounds on the surface of PJBC. It aids in facilitating the qualitative evaluation of materials and interpreting any existent organic groups. The biochar derived from *Prosopis Juliflora* biomass waste was analyzed using FTIR analysis. Using a kiln, pyrolysis entails burning biomass at a specific temperature to dissolve the chemical bonds. The different kind of functional groups contained in the adsorbent material were determined via FTIR analysis^49^. Figure 13 exhibits the amide, aldehyde, ester, anhydride, and alkyl functional group vibration in the 400–4000 cm^− 1^ wavelength range made of Perkin Elmer machine for solids and liquids, as well as the phenolic, alcoholic hydroxy bond (OH), ketonic (C = O), carboxylic (COO), and many other vibrations.

**Table 9:** Different functional groups of PJBC.

Wave number (cm^− 1^)		Organic groups and their nature	Chemical formation
Theoretical range	Experimental outputs		
3400–3800	3434.52, 3671, 3619	C–H & stretching	Alcoholic, phenolic
2800–3000	2849.85, 2911.44	C–H & stretching	Alkanes
1680–2700	2017.24	C–H & bending	Aromatic
1250–1650	1617.32, 1387.55, 1273.24	C=C & stretching	Alkenes
1200–1000	1097.91	C–N & stretching	Aromatic amine
1000−500	776.28, 571.34	–COOH & stretching	Carboxylic

The results showed in Table 9 the presence of several functional groups, including a strong C-H stretching vibration at 3434.52 cm^− 1^ and a vinyl C-H bonding of alkanes at 2849.85 cm^− 1^. Alcohol and phenol groups were also detected at 3671 cm^− 1^ and 3619 cm^− 1^, respectively. Broad peaks indicated the presence of alkane stretching and aromatic bending at 2911.44 cm^− 1^ and 2017.24 cm^− 1^. An alkene stretching group was found at 1617.32 cm-1, while the alkenes group was also observed around 1387.55 and 1273.24 cm^− 1,^ respectively. The fingerprint area showed indications of an aromatic amine group at 1097.91 cm^− 1^ and carboxylic -COOH compounds at 776.28 and 571.34 cm^− 1^, enhancing the catalytic activity of the PJBC^21^. It should be noted that while the peaks at 2017.24 and 1387.55 cm^− 1^ were narrow, suggesting the presence of the aromatic and alkenes group, other peaks are broad, showing the weaker strength in the PJBC: Carboxyl and hydroxyl groups formed during pyrolysis, while an acidic function group was eliminated.

**Fig. 14:**
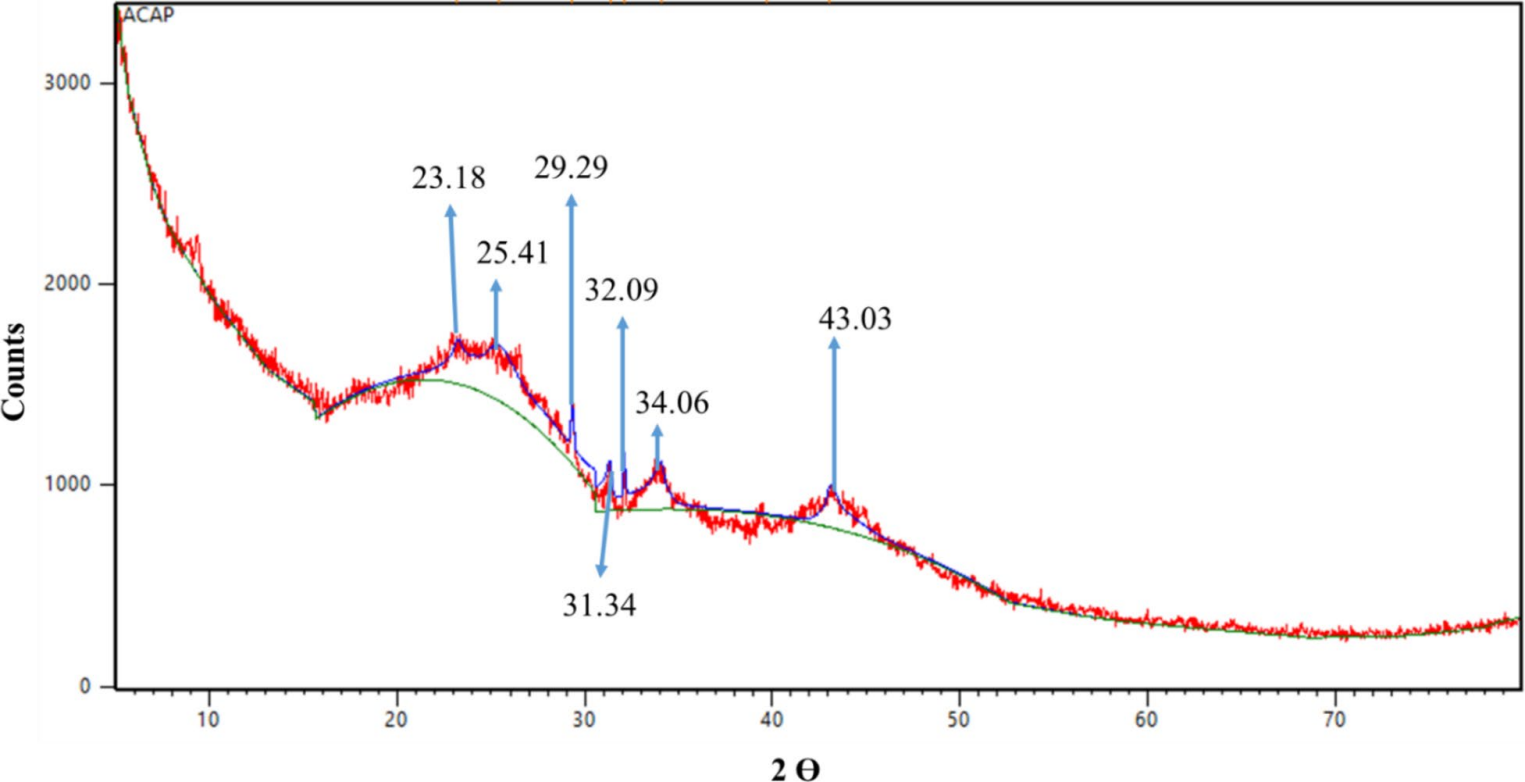
XRD pattern of PJBC.

The analysis of PJBC using X-ray diffraction (XRD) confirmed the crystalline nature of the synthesized carbon from the *Prosopis Juliflora* plant^50^. A Bruker diffractometer was used to identify XRD patterns and wavelength (λ) of 1.647 Å with CuKα radiation to identify and quantify the phase. The XRD pattern of PJBC exhibited in Fig. 14 implies the various diffraction peaks at 2θ angles of 23.18, 25.41, 29.29, 31.34, 32.09, 34.06, and 43.03°, respectively. These peaks matched the JCPDS no. 81–0792, indicating that PJBC has a body-centred cubic (BCC) structure with no impurities. The peak point 29.29°, with the greatest peak value, was employed to determine the PJBC crystallite size, 26.78 nm, using Debye-Scherrer’s Eq. (4)^51^. The XRD analysis confirmed that PJBC synthesized from the *Prosopis Juliflora* biomass waste is a pure and crystalline material with a BCC structure.

4 $$\:Crystalline\:size\:\left(nm\right)=\frac{k\times\:\lambda\:}{\beta\:\times\:cos \theta}$$

Where k = Scherrer constant (0.89 to 1); λ = wavelength (Å) of the PJBC; β = X-ray diffraction peaks at FWHM; θ = Braggs angle.

#### EDX, BET and XPS analysis of PJBC

**Fig. 15:**
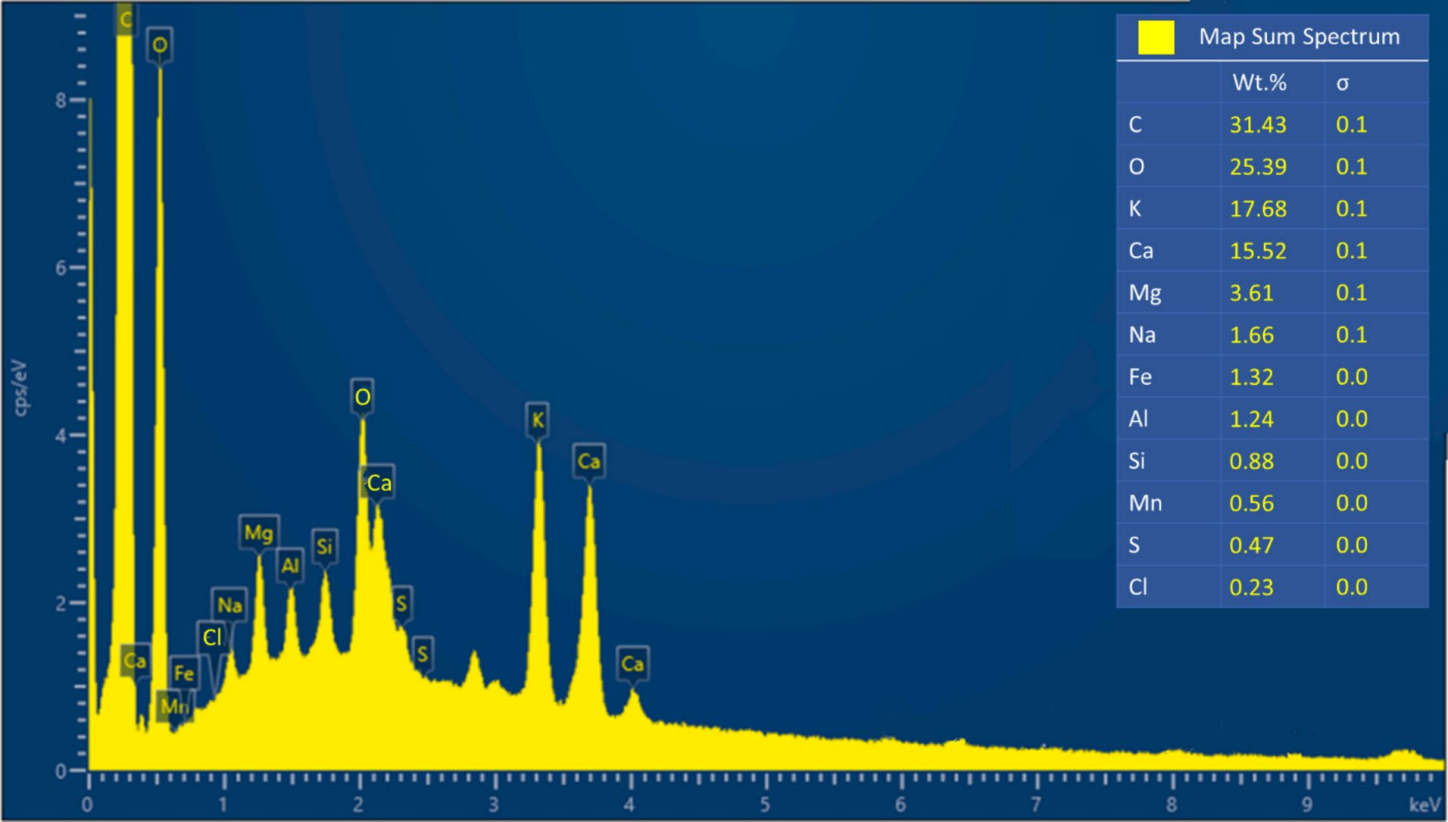
EDX of PJBC.

An examination of the PJBC sample utilizing EDX covered the existence of various inorganic components, specifically Potassium (K), Calcium (Ca), Magnesium (Mg), and Sodium (Na), as shown in Fig. 15. The fast pyrolysis process is responsible for the existence of K, Ca, Mg, and Na in biochar. These mineral ions can offer macro and micronutrients to the soil, promote plant growth, and decrease NO_x_ emissions. Moreover, these elements are readily soluble in water, ensuring their safety for plants and the environment^52^. However, the biochar sample contains very minimal silica, which would have enhanced pollen fertility in plants if present. This PJBC sample is a promising option for catalytic activity due to the presence of very rich Oxygen (O), Potassium (K), Calcium (Ca), Magnesium (Mg), and traces of Sodium (Na) to accelerate the reaction in trans-esterification and soil improvement or conditioning.

**Fig. 16:**
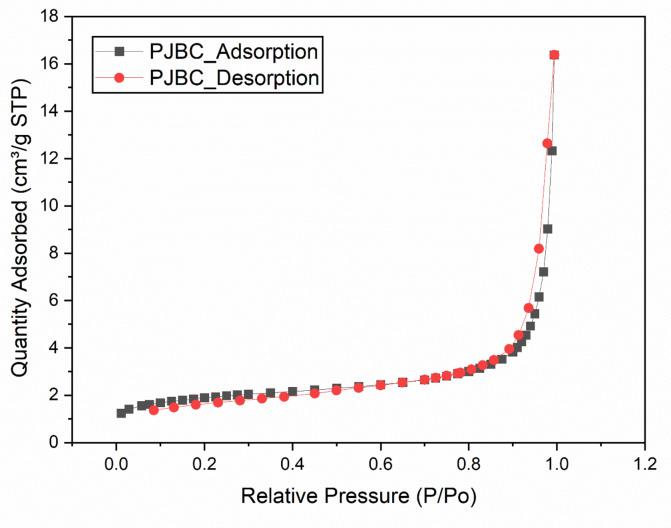
N_2_ adsorption-desorption isotherm of the PJBC-based heterogeneous catalyst.

The specific surface area, pore volume and average pore size of the PJBC-based catalyst were calculated from the N_2_ adsorption-desorption isotherm in Brunauer-Emmett-Teller (BET) analysis, as shown in Fig. 16. It showed that the single point surface area at P/Po = 0.29875 is 6.212 m^2^/g, and the pore volume for the same is 0.02532 cm^2^/g. Similarly, the BET-specific surface area for PJBC-based catalyst is 6.281 m^2^/g, and this lower volume confirmed the enhancement of catalytic activity for biodiesel production^53^. Also, the Barrett-Joyner-Halenda (BJH) adsorption average pore diameter for the PJBC catalyst is 28.01 nm, and the desorption average pore diameter for the same is 23.63 nm. According to the International Union of Pure and Applied Chemistry (IUPAC) isotherm type IV, the obtained pore diameters are within the limit of 2 nm to 50 nm, ensuring the characteristic of mesoporous materials^54^. It provides ample active sites for catalytic reactions.

**Fig. 17:**
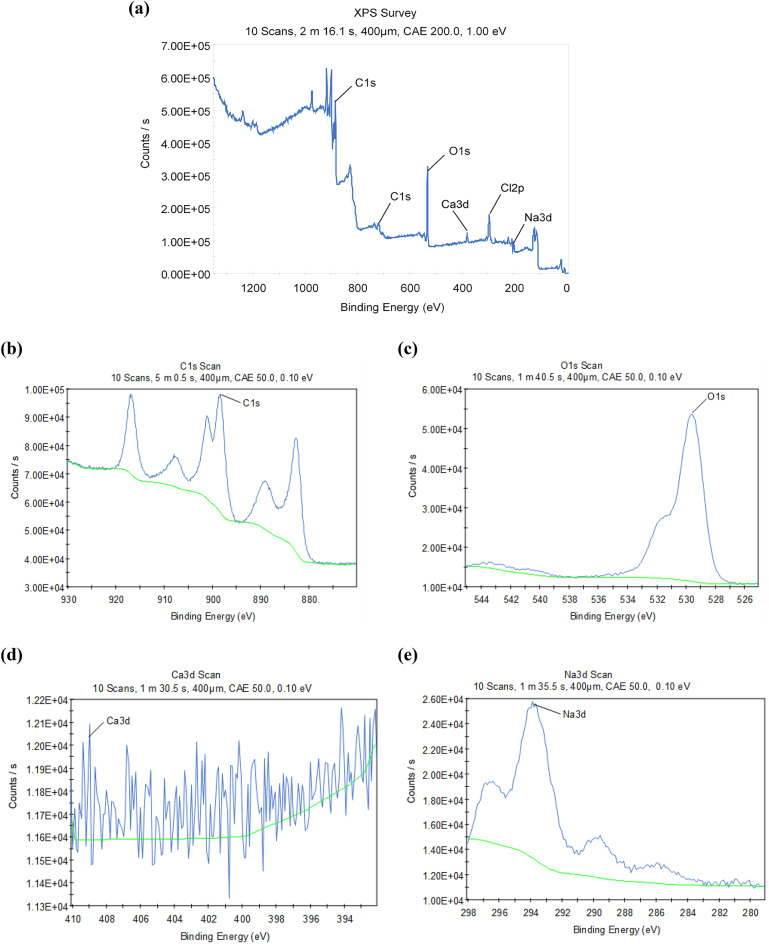
XPS of (**a**) Complete survey results, (**b**) C1s scan plot, (**c**) O1s scan plot, (**d**) Ca3d scan plot and (**e**) Na3d scan plot.

The experiments using X-ray photoelectron spectroscopy (XPS) were carried out to determine the oxidation states on the elemental surfaces of the PJBC catalyst. A survey’s XPS spectrum, C1s, O1s, Ca3d, Cl2p, and Na3d, are depicted in Fig. 17 (a). The whole spectrum comprises the conspicuous, distinctive peaks that originally emerged at the binding energy of C1s @ 881.36 eV, O1s @ 530.12 eV, Ca3d @ 409.01 eV, Cl2p @ 294.43 eV, and Na3d @ at 200.14 eV, respectively. Two peaks at 881.36 eV and 743.64 eV were visible in the PJBC catalyst’s high-resolution C1s spectrum in Fig. 17 (b), which suggested that many functional groups, such as hydroxyl, carboxyl, and graphitic sp^2^ carbon, were linked to carbon in the PJBC^55^. Figure 17 (c) indicates the O1s scan of PJBC; the peak @ 530.12 eV binding energy ensured the availability of oxygen (O) molecules. The binding energy peak for the 3d spectra, located at 409.01 eV in Fig. 17 (d), provides information on the conformation of Ca present on the PJBC catalyst surface. Finally, a trace amount of sodium (Na) in PJBC was found through a Na3d scan (Fig. 17 (e)), supporting the solid base catalytic activity in the transesterification process^33^.

### Characterization studies on WTSB

This section covers the characterization studies conducted on WTSB to determine their chemical compositions and functional groups. It also compares the physico-chemical characteristics and yield rates of neat WTSB with those reported in previous literature.

#### GC-MS analysis on WTSB

GC-MS (Gas chromatograph coupled with mass spectrometer) is an analytical method performed to identify and quantify the WTSB’s chemical composition. In this test, the WTSB is vaporised and injected into the gas chromatograph, which separates the WTSB into individual components based on properties such as boiling point and polarity. These separated components were then introduced to the mass spectrometer, which was ionized into small charged particles using an FID detector. Then, it analyses the charged particles based on the mass-to-charge ratio, producing a mass spectrum that provides information on the number of individual components in the WTSB. The amount of each compound present in a WTSB is determined by comparing it with the resultant mass spectrum to the standard spectra. There are many peaks that are visible, hence the increased presence of ester compounds, which is listed in Table 10. The number of components contained in the WTSB is indicated by the peak’s % area. The maximum percentage of WTSB conversion from WTSO using PJBC as catalyst was primarily confirmed by the presence of Dodecanoic acid Methyl Ester (10.14%), Hexadecanoic acid Methyl Ester (12.71%), 9,12-octadecadienoic acid (Z, Z)-Methyl Ester (21%), and 9-octadecenoic acid Methyl Ester (39.84%) in the WTSB chromatogram.

**Table 10:** Major chemical compounds present in the WTSB.

Peak area (%)	Chemical formula	Chemical compounds	Fatty acid	Nature of fatty acid
10.14	C_13_H_26_O_2_	Dodecanoic acid, methyl ester	Lauric acid	Saturated
5.16	C_15_H_30_O_2_	Tetradecanoic acid, methyl ester	Myristic acid	Saturated
12.71	C_17_H_34_O_2_	Hexadecanoic acid, methyl ester	Palmitic acid	Saturated
21	C_19_H_34_O_2_	9,12-octadecadienoic acid (Z, Z)-, Methyl Ester	Linoleic acid	Polyunsaturated
39.84	C_19_H_36_O_2_	9-octadecenoic acid, methyl ester	Oleic acid	Monounsaturated
6.37	C_21_H_42_O_2_	Eicosanoic acid, methyl ester	Arachidic acid	Saturated
2.23	C_23_H_44_O_2_	13-Docosenoic acid, methyl ester	Erucic acid	Monounsaturated

#### FTIR analysis on WTSB

**Fig. 18:**
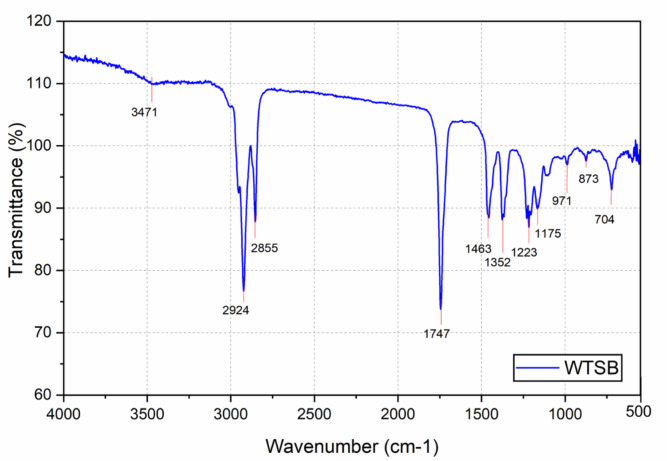
FTIR spectra of WTSB.

Fourier Transform Infra-Red (FTIR) spectroscopy is an analytical test carried out to identify and characterize the functional groups in WTSB. FTIR analysis works for a range of wavelengths in the infrared spectrum, which are transmitted through the WTSB. The WTSB’s ability to transmit the IR ray’s energy at different wavelengths determines the presence of different functional groups in the WTSB’s chemical composition. A graph (Fig. 18) is created by plotting the corresponding wavenumber (cm^− 1^) on the X-axis and the transmittance (%) of the WTSB on the Y-axis. Two bands within the mid-IR spectrum range from 4000 cm^− 1^ to 500 cm^− 1^. The regions of the functional group (4000 cm^− 1^ to 1500 cm^− 1^) and fingerprint (1500 cm^− 1^ to 500 cm^− 1^). A noticeable peak at 3471 cm^− 1^ demonstrates the stretching vibration with weak absorption intensity of the hydroxyl (-OH) functional group, implying that WTSB substances contain phenols or alcohols. Asymmetric and symmetric stretching vibrations with substantial alkane absorption intensity are shown by the peaks at 2924 cm^− 1^ and 2855 cm^− 1^, respectively. These vibrations are thought to reflect the -CH₂ functional groups in WTSB resulting from transesterification. The appearance of a prominent absorption intensity peak at 1747 cm^− 1^ corresponding to a C = O stretching vibration validates the production of esters, such as mono-alkyl esters, in WTSB structures. The peak at 1463 cm^− 1^ is associated with bending vibrations, such as CH₂ bending. Similarly, the C-O-C functional group formed at a sharp point of 1352 cm^− 1^, resulting in ester groups with medium absorption intensity levels. A small peak at 704 cm^− 1^ represents the out-of-plane bending (rocking) vibration of C-H bonds, indicating the presence of aromatic compounds in WTSB. Based on the results of the FTIR spectra, the existence of oxygen levels in WTSB is ensured. It could be proven to be an effective replacement for the present diesel fuel^56^.

#### Comparison of PJBC catalyst-derived WTSB with existing literature

The WTSB produced by the PJBC catalyst in this work is compared to the outcomes of other biochar-based catalysts that have been documented and used to make biodiesel in Table 11. This comparison considers the biochar feedstock, the technology used for synthesizing biochar-based catalysts, the biodiesel feedstock and its optimized parameters to obtain maximum biodiesel yield through the transesterification method. It was discovered that all of the biochar feedstocks used are biomass waste, and the most popular methods for creating catalysts are pyrolysis and calcination. Using these catalysts, biodiesel is produced from a variety of second-generation feedstocks that are mostly derived from biomass waste. All of the previously chosen parameters from various literature sources are more in line with the present study. However, the greater WTSB yield (97.42%) attained using PJBC catalysts is more in line with existing literature findings, which indicate that biodiesel yields vary from 90 to 100% when biochar-based catalysts are used to produce biodiesel from various biomass waste feedstocks.

**Table 11:** Comparing PJBC with other documented biochar-based catalysts used for biodiesel production.

Biochar feedstock	Technique for synthesizing catalyst	Biodiesel feedstock	Optimum transesterification parameters	Biodiesel yield (%)	Reference
*Prosopis Juliflora* branches	Slow pyrolysis (790 °C, 60 mg and 60 min)	Waste *Trichosanthes cucumerina* seed bio-oil (WTSO)	PJBC catalyst (4 wt%), methanol to WTSO molar proportion (13:1), reaction temperature (60 °C) and time (60 min)	97.42	This study
Rice husk	Calcination process (450 °C, 20 mg and 180 min)	Used cooking oil (USO)	RHC/K_2_O-20%/Ni-5% catalyst (4 wt%), methanol/UCO molar ratio (12:1), temperature (65 °C) and reaction time (120 min)	98.20	^12^
Banana peels	Calcination process (700 °C, 40 mg and 240 min)	Napoleon’s plume seed oil (NPSO)	Banana peel ash catalyst (3 wt%), methanol/NPSO molar ratio (8:1), reaction temperature (65 °C) and time (70 min)	98.50	^57^
*Heteropanax fragrans* branches	Calcination process (550 °C, 20 mg and 120 min)	Jatropha curcas oil (JCO)	Heteropanax fragrans catalyst (7 wt%), methanol/JCO molar ratio (12:1), operating temperature (65 °C) and time (65 min)	97.75	^58^
Ripe banana (*Musa balbisiana Colla*) fruit peels	Calcination process (700 °C, 50 mg and 240 min)	Waste cooking oil (WCO)	*Musa balbisiana Colla* peel ash catalyst (2 wt%), methanol/WCO ratio (6:1), temperature (60 °C) and time duration (180 min)	100	^59^
Pineapple (*Ananas comosus*) leaves	Calcination process (600 °C, 30 mg and 120 min)	Soybean oil	Pineapple leaves ash catalyst (5 wt%), methanol/soybean oil ratio (40:1), reaction temperature (60 °C) and time (30 min)	98	^60^
Potato (*Solanum tuberosum* *L.)* peels	Fixed bed pyrolysis (500 °C) with calcination (700 °C, 20 g and 180 min)	Soybean waste cooking oil (SWCO)	Potato peel catalyst (3 wt%), methanol/SWCO ratio (9:1), operating temperature (60 °C) and time (120 min)	97.50	^61^
Cotton black liquor	Spray drying and fast carbonization (700 °C)	Waste frying oil (WFO)	Cotton black liquor catalyst (6.5 wt%), methanol/WFO ratio (7.7), process temperature (65 °C) and duration (120 min)	91.50	^62^
Coconut *(Cocos nucifera)* husk	Low sulfonation (120 °C) and carbonization (500 °C, 60 min)	Kanuga oil	Coconut husk catalyst (ACH-SO_3_H) (5 wt%), methanol/kanuga oil molar ratio (40), operating temperature (55 °C) and time (80 min)	98.99	^53^

### Environmental impact analysis of WTSB

This section evaluates the neat WTSB and WTSB/diesel blends in a DI diesel engine under different load circumstances to compare their performance, combustion, and emission characteristics with those of existing diesel fuel.

#### Brake thermal efficiency

**Fig. 19:**
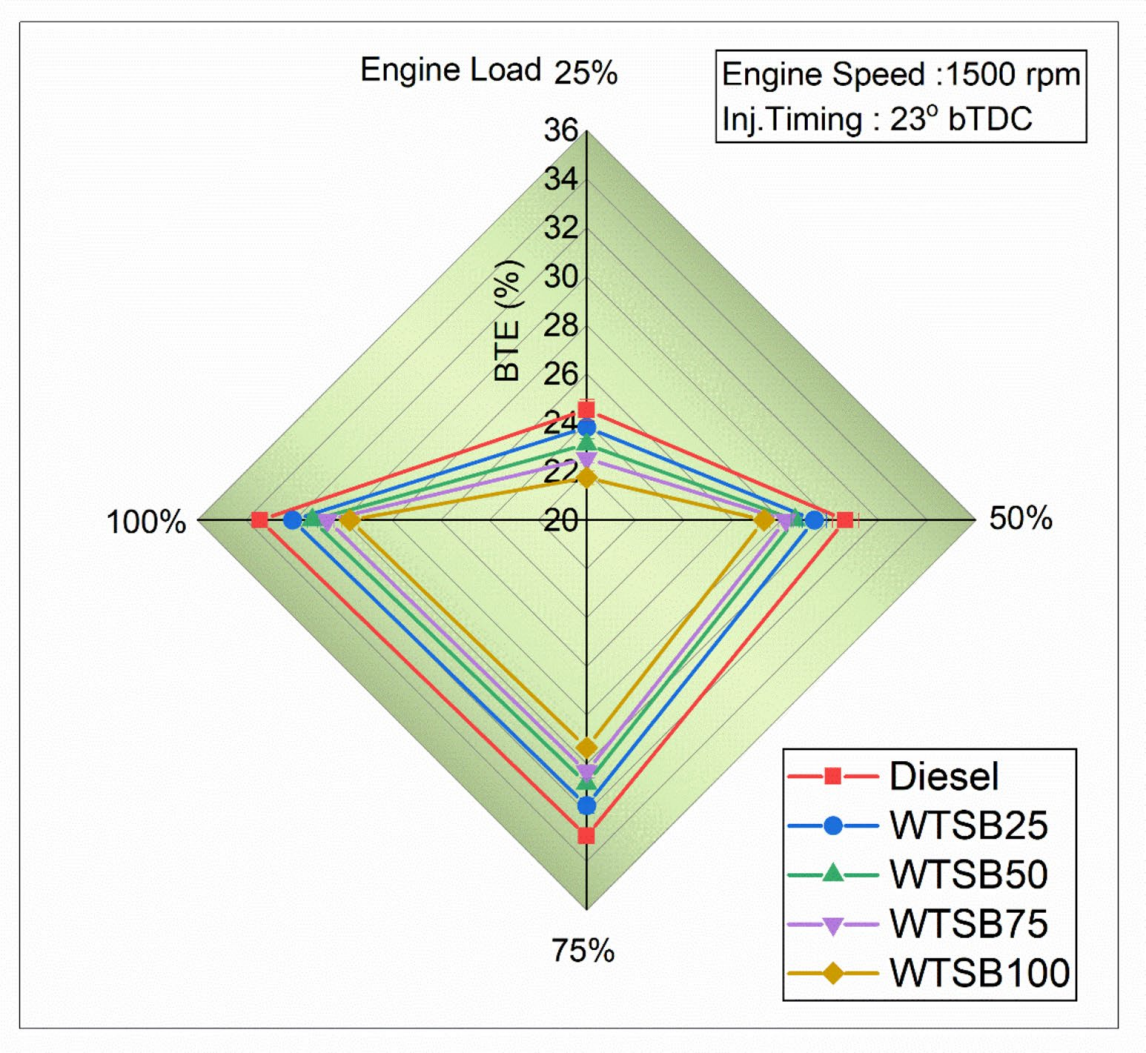
Changes in BTE (%) for diesel and WSTB blends with engine load.

The changes in brake thermal efficiency (BTE) with engine load for dissimilar fuels of diesel, WTSB25, WTSB50, WTSB75 and WTSB100 are depicted in Fig. 19. The BTE evaluates the effectiveness of fuel combustion within the domed combustion cavity and its conversion into usable energy^63^. As shown in Fig. 19, BTE increases progressively with rising engine load for all test fuel blends. However, increasing the WTSB blend ratio in diesel fuel results in a declining BTE trend across all engine loads. This decline is attributed to the reduced heating value and higher fluid viscosity, which impair fuel dispersion and vaporization^64^. As a result, higher WTSB blends lead to reduced BTE. The observed BTE at 100% engine load was 33.43% for diesel, 32.07% for WTSB25, 31.27% for WTSB50, 30.66% for WTSB75, and 29.73% for WTSB100. The WTSB25 blend has the closest BTE to diesel fuel out of all the WTSB mixes. Its thermophysical characteristics, which closely resemble those of existing diesel fuel, lead to a greater BTE. Prabhakaran et al.^65^ observed a similar trend in their BTE findings from *Azolla-*based biofuels. They highlighted that the reduced calorific content and increased viscosity of the fluid contribute to a lower rate of A/F combination, inadequate dispersal of *Azolla* biodiesel, and evaporation, leading to reduced BTE.

#### Brake specific energy consumption

**Fig. 20:**
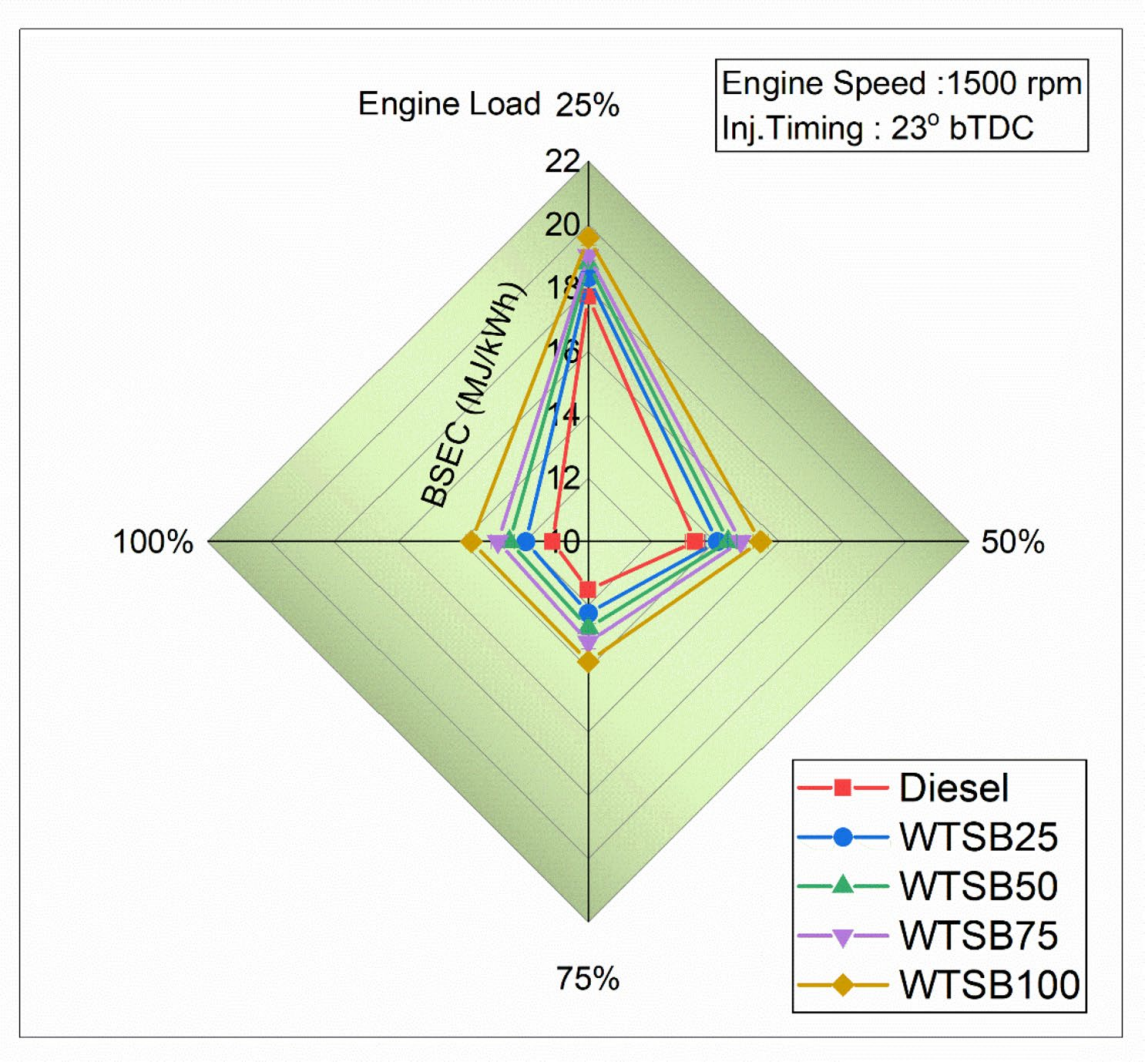
Changes in BSEC (MJ/kWh) for diesel and WSTB blends with engine load.

Figure 20 shows that the brake-specific energy consumption (BSEC) decreases with increasing engine load from 25 to 100% for all WTSB blends. However, as the WTSB blend ratio in existing diesel fuel increases, there is a notable rise in BSEC across all engine load conditions. This results from WTSB’s reduced heating value, which necessitates a higher fuel injection rate during engine operation to sustain steady speed. At 100% engine operation, the BSEC values are recorded as 11.13 MJ/kWh for diesel, 12.25 MJ/kWh for WTSB25, 12.49 MJ/kWh for WTSB50, 12.85 MJ/kWh for WTSB75, and 13.69 MJ/kWh for WTSB100. At lower engine loads (25%, 50% and 75%), BSEC values are elevated due to reduced in-cylinder temperatures and inadequate fuel mixing. These factors lead to incomplete combustion, thereby increasing the BSEC^66^. The WTSB25 blend consistently exhibits lower BSEC compared to other WTSB blends across all load conditions. This can be attributed to its higher heating value, along with its lower density and kinematic viscosity, which contribute to reduced BSEC. According to Prasanna Raj Yadav et al.^67^, when compared to larger biofuel blends, the B25 biofuel mixture exhibited the least energy consumption at 100% load. This is attributed to its structural and thermal characteristics, which are more closely aligned with those of neat diesel fuel.

#### In-cylinder pressure

**Fig. 21:**
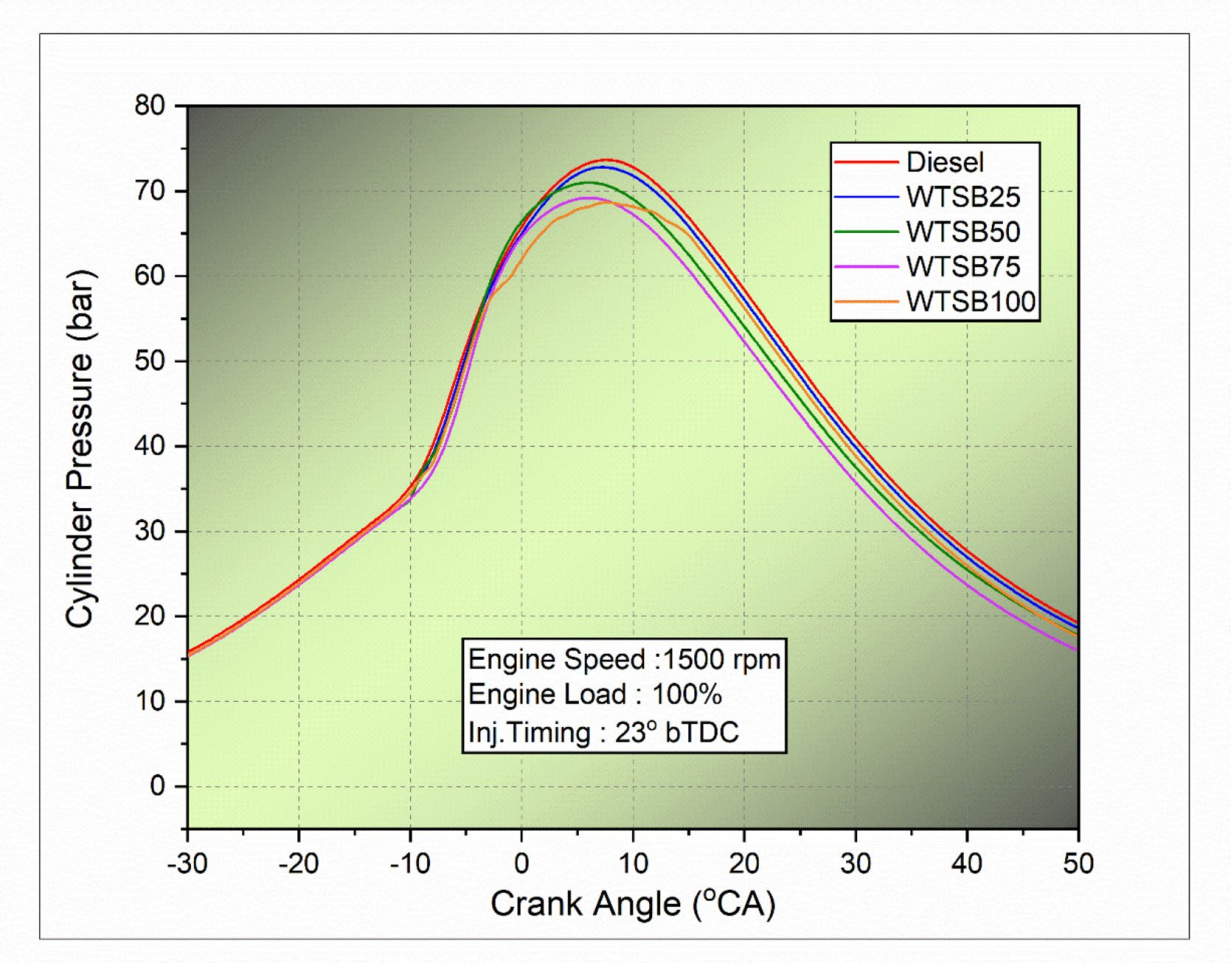
Changes in in-cylinder pressure (bar) for diesel and WSTB blends with crank angle.

In-cylinder pressure is a key factor in assessing the maximum pressure during fuel combustion. Optimal combustion is achieved when the peak cylinder pressure occurs near the top dead center (TDC), enhancing thermal power conversion into effectual energy output^35^. Figure 21 displays the changes in in-cylinder pressure across different crank angles for diesel, WTSB25, WTSB50, WTSB75, and WTSB100 blends. As the WTSB blend ratio in diesel fuel increases, a reduction in in-cylinder peak pressure is observed. This downward trend is mainly attributed to the higher viscous nature, increased density, and reduced heating value of the WTSB blends, which adversely affect the rate of WTSB mixing, atomization, volatilization, and spray reach during the unregulated ignition^35^.

Figure 21 shows that diesel fuel, with a peak in-cylinder pressure of 73.62 bar, has the highest value compared to the WTSB blends, which have peak pressures of 72.37 bar (WTSB25), 70.48 bar (WTSB50), 68.07 bar (WTSB75), and 66.58 bar (WTSB100). This higher peak pressure in diesel is attributed to its reduced fluid viscosity (2.71 mm²/s), lower mass density (836 kg/m³), and higher energy content (42.50 MJ/kg), which improve fuel spray characteristics and result in more efficient combustion. The WTSB100 blend showed reduced cylinder gas pressure compared to other WTSB blends. This is likely because of its higher kinematic viscosity (3.86 mm²/s) and lower heating value (39.12 MJ/kg), which may have resulted in a lower air-fuel relation and inadequate fuel fragmentation at ignition. Raman et al.^68^ reported an equivalent pattern of in-cylinder pressure when using various biodiesel blends. Their findings showed that increasing the proportion of biodiesel (from 25 to 100%) led to a decrease in peak cylinder pressure. This behaviour is mainly due to biodiesel’s higher cetane number, which shortens the ignition delay period and thus limits the maximum pressure rise.

#### Heat release rate

The heat release rate (HRR) is an essential parameter for evaluating the ignition operation in a DI diesel engine. It quantifies the conversion of chemical energy released during fuel combustion into thermal energy^69^. The heat release is calculated using the first law of thermodynamics, as expressed in Eq. (5).

5 $$\:\frac{{\text{d}\text{Q}}_{\text{h}\text{r}\text{r}}}{\text{d}{\uptheta\:}}=\left(\frac{{\upgamma\:}}{{\upgamma\:}-1}\right)\times\:\text{P}\left({\uptheta\:}\right)\times\:\left(\frac{\text{d}\text{V}}{\text{d}{\uptheta\:}}\right)+\left(\frac{1}{{\upgamma\:}-1}\right)\times\:\text{V}\left({\uptheta\:}\right)\times\:\left(\frac{\text{d}\text{P}}{\text{d}{\uptheta\:}}\right)$$

Here, $\:\frac{{\text{d}\text{Q}}_{\text{h}\text{r}\text{r}}}{\text{d}{\uptheta\:}}\:\:$denotes the heat release rate in J/^o^CA, γ represents the ratio of specific heats c_p_/c_v_ (γ = 1.3), P signifies the in-cylinder pressure in bar, and V is the volume within the cylinder, measured in cubic meters (m³).

**Fig. 22:**
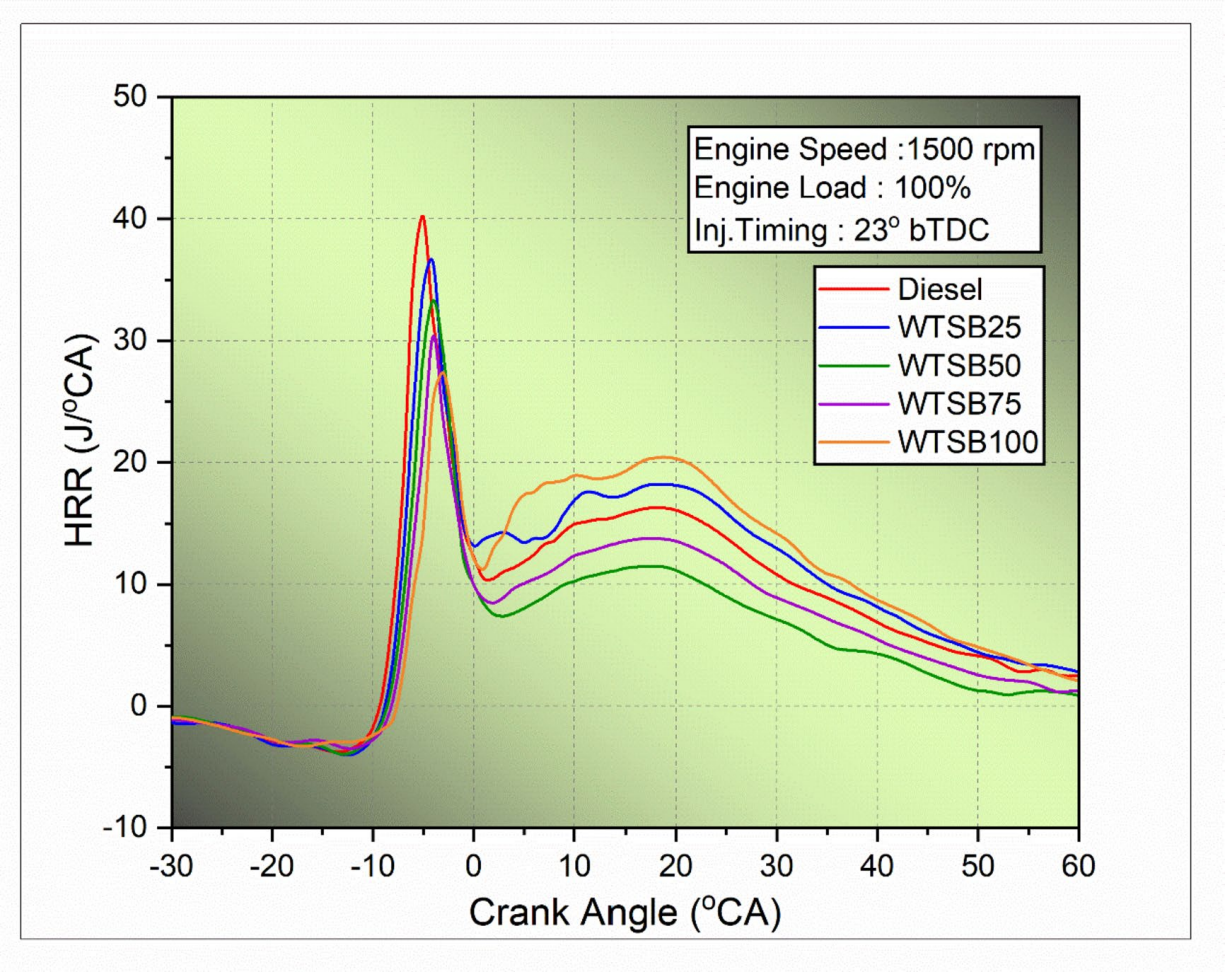
Changes in HRR (J/^o^CA) for diesel and WSTB blends with crank angle.

Figure 22 illustrates the variation in heat release rate (HRR) for diesel and various WTSB blends with respect to crank angle under 100% engine load conditions. The HRR curve for WTSB100 and its blends shows a deceleration compared to diesel during the premixed combustion phase. This behavior is attributed to the higher cetane rating of WTSB mixes, which shortens the ignition lag and accelerates the burning process, thereby reducing the elevated HRR and influencing peak pressure more effectively than diesel fuel^70^. Notably, neat diesel fuel had a higher HRR of 40.21 J/°CA. In comparison, the HRR values for WTSB blends were significantly lower: WTSB25 at 36.33 J/°CA, WTSB50 at 32.51 J/°CA, WTSB75 at 30.42 J/°CA, and WTSB100 at 27.38 J/°CA, all under 100% engine load. Despite this, the oxygen content in the WTSB blends leads to slower combustion, which results in a higher HRR during the diffusion combustion phase. This demonstrates that a larger proportion of the combustion for neat WTSB and its blends occurs in the diffusion period, producing more HRR compared to the premixed combustion period^71^. Among the WTSB blends, WTSB25 exhibited a HRR comparable to that of diesel fuel in both the initial and subsequent phases of combustion. This similarity is likely due to WTSB25’s higher heating value and oxygen content, which improve the air-fuel mixing rate and fuel droplet atomization, thereby enhancing combustion efficiency in the engine. Ramalingam et al.^72^ carried out comparable research and discovered that the B20 blend exhibited an elevated HRR similar to that of existing diesel. This equivalence in peak HRR was due to the B20 blend’s properties being identical to those of neat diesel.

#### Specific unburned hydrocarbon emission

**Fig. 23:**
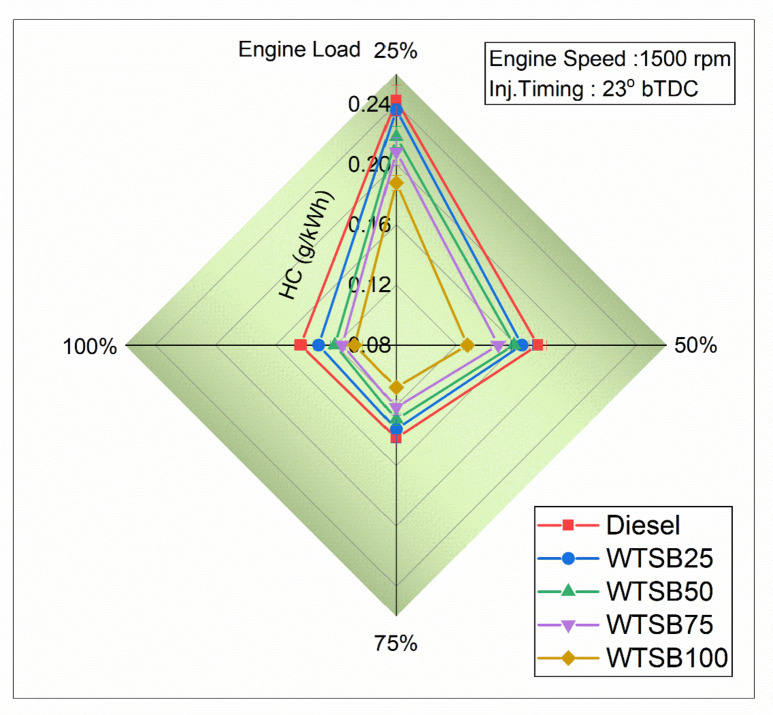
Changes in S_HC_ (g/kWh) emission for diesel and WSTB blends with engine load.

Figure 23 illustrates the specific hydrocarbon (S_HC_) emission profiles of the test engine operating with diesel and WTSB blends across 25%, 50%, 75% and 100% engine loads. In general, S_HC_ emissions result from the partial combustion of WTSB within the engine cylinder. This incomplete combustion can be attributed to factors such as inadequate or excessive mixing of WTSB with air during the ignition delay period, fuel spray collision on the engine cylindrical boundaries, and injector fluid capacity^29^. Figure 23 showed that WTSB mixes consistently yield less S_HC_ emissions than diesel fuel across all operating conditions. This is due to the oxygen content in WTSB, which enhances combustion efficiency and reduces S_HC_ formation. A significant reduction in S_HC_ emissions was observed for all WTSB blends during 100% engine load operation. Specifically, the S_HC_ emissions were 0.143 g/kWh for existing diesel, 0.131 g/kWh for WTSB25, 0.12 g/kWh for WTSB50, 0.116 g/kWh for WTSB75, and 0.107 g/kWh for WTSB100. The S_HC_ emissions showed a reduction of 8.39%, 16.08%, 18.92%, and 25.17% for the WTSB25, WTSB50, WTSB75, and WTSB100 blends, respectively, compared to diesel fuel. This reduction is attributed to the higher oxygen content and cetane value of the WTSB blends, which increase combustion chamber gas temperature, shorten the ignition lag, and encourage more efficient ignition, thereby reducing S_HC_ formation^73^. Raman et al.^68^ observed the same pattern of S_HC_ emissions with increasing rapeseed oil biodiesel (RSOB) blend and engine load. The study confirmed that the oxygen molecules in RSOB enhance fuel combustion through proper combining, sparking, and dispersing, which minimizes S_HC_ emissions compared to existing diesel fuel.

#### Specific carbon monoxide emission

**Fig. 24:**
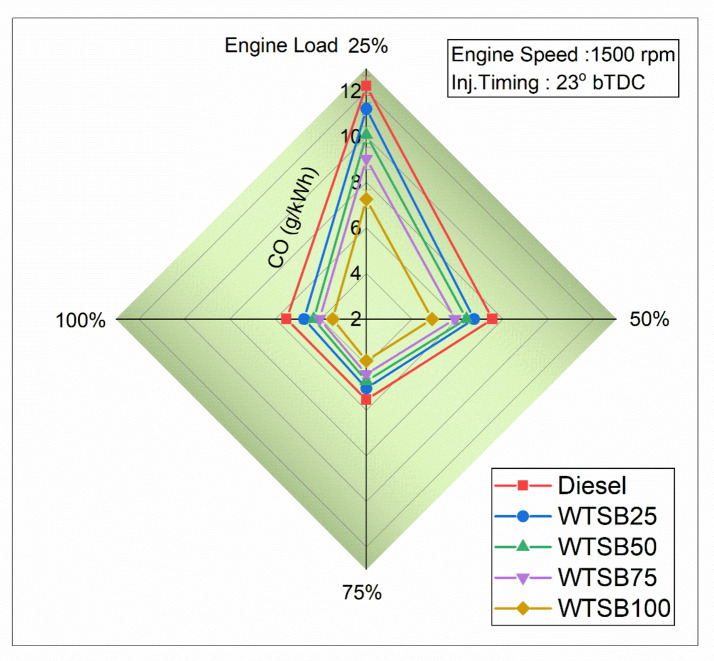
Changes in S_CO_ (g/kWh) emission for diesel and WSTB blends with engine load.

Carbon monoxide formation in diesel engine operation is primarily driven by an oxygen deficiency in fuel-rich regions, lower air-fuel proportions, and low-temperature burning of fuel^74^. Figure 24 depicts the variation in specific CO (S_CO_) emissions with increasing engine load across different WTSB blends. The highest percentage of S_CO_ emissions was recorded at 25% engine load for all WTSB blends, compared to 100% engine load. This is attributed to the lower in-cylinder gas temperature at 25% load, leading to incomplete combustion of the WTSB blends that contribute to an increase in S_CO_ emissions. At 100% engine load, S_CO_ emissions were reduced by 13.97% for WTSB25, 21.78% for WTSB50, 26.32% for WTSB75, and 36.84% for WTSB100 compared to diesel fuel. This reduction is attributed to the oxygen content in the WTSB chemical compounds, which promotes the oxidation of CO into carbon dioxide (CO_2_), thereby reducing the production of S_CO_^74^. Balasubramanian et al.^41^ discovered that changing waste cooking oil blends in DI engine operation reduced S_CO_ emissions, with higher blends emitting less S_CO_ due to increased O_2_ content. This oxygen promotes CO oxidation to CO_2_, leading to lower S_CO_ emissions at 100% engine load. These findings are also supported by Prasanna Raj Yadav et al.^67^, who reported similar results in their research.

#### Specific nitric oxide emission

**Fig. 25:**
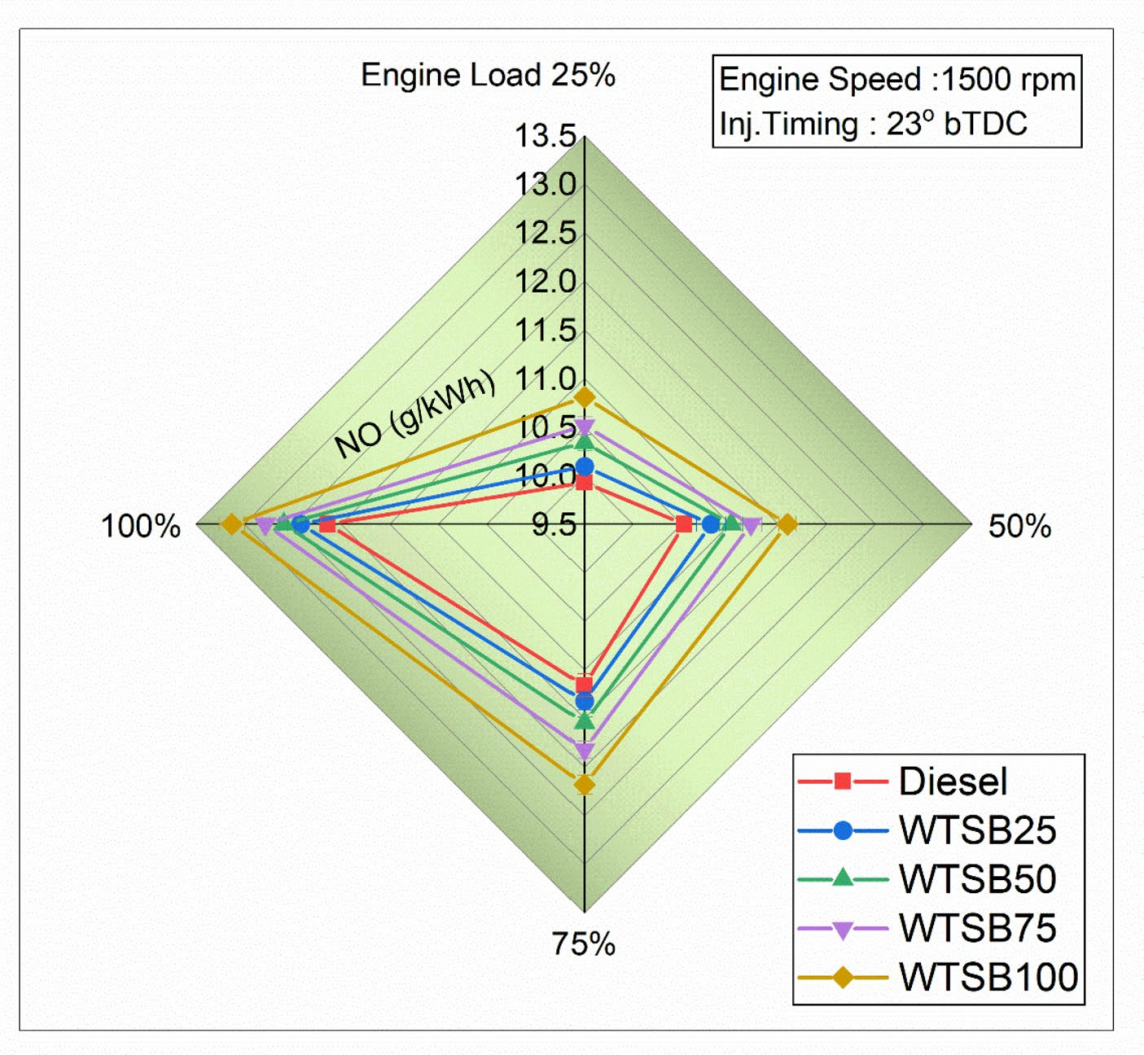
Changes in S_NO_ (g/kWh) emission for diesel and WSTB blends with engine load.

Figure 25 shows that increasing the WTSB blend ratio in diesel fuel results in a higher formation of specific nitric oxide (S_NO_) emissions across all engine loads compared to neat diesel fuel. S_NO_ formation primarily depends on higher in-cylinder temperatures, prolonged dwell periods, and oxygen delivery in the fuel components^24^. Consequently, a diesel engine operating at 100% load generates the highest temperatures for the gas within the cylinder, leading to higher S_NO_ emissions in both neat WTSB and its blends. This is due to the increased kinematic viscosity, volume, and O_2_ content of WTSB, which raises in-cylinder temperatures and enhances S_NO_ formation. However, diesel fuel produces fewer S_NO_ emissions due to its lower oxygen concentration. At 100% engine load operation, S_NO_ emissions are recorded as 12.04 g/kWh for existing diesel, 12.42 g/kWh for WTSB25, 12.59 g/kWh for WTSB50, 12.78 g/kWh for WTSB75, and 13.13 g/kWh for WTSB100. Considering different WTSB blends, WTSB25 has the lowest S_NO_ emissions, primarily due to its lower air-fuel (A: F) ratio and higher calorific value. Ramakrishnan et al.^75^ observed that S_NO_ emissions rise with the increasing percentage of biodiesel blend compared to existing diesel fuel. They explained this trend by noting that the higher O_2_ components in biodiesel lead to increased combustion of test fuel temperatures, which in turn causes S_NO_ emissions to increase across all test blends.

#### Smoke opacity emission

**Fig. 26:**
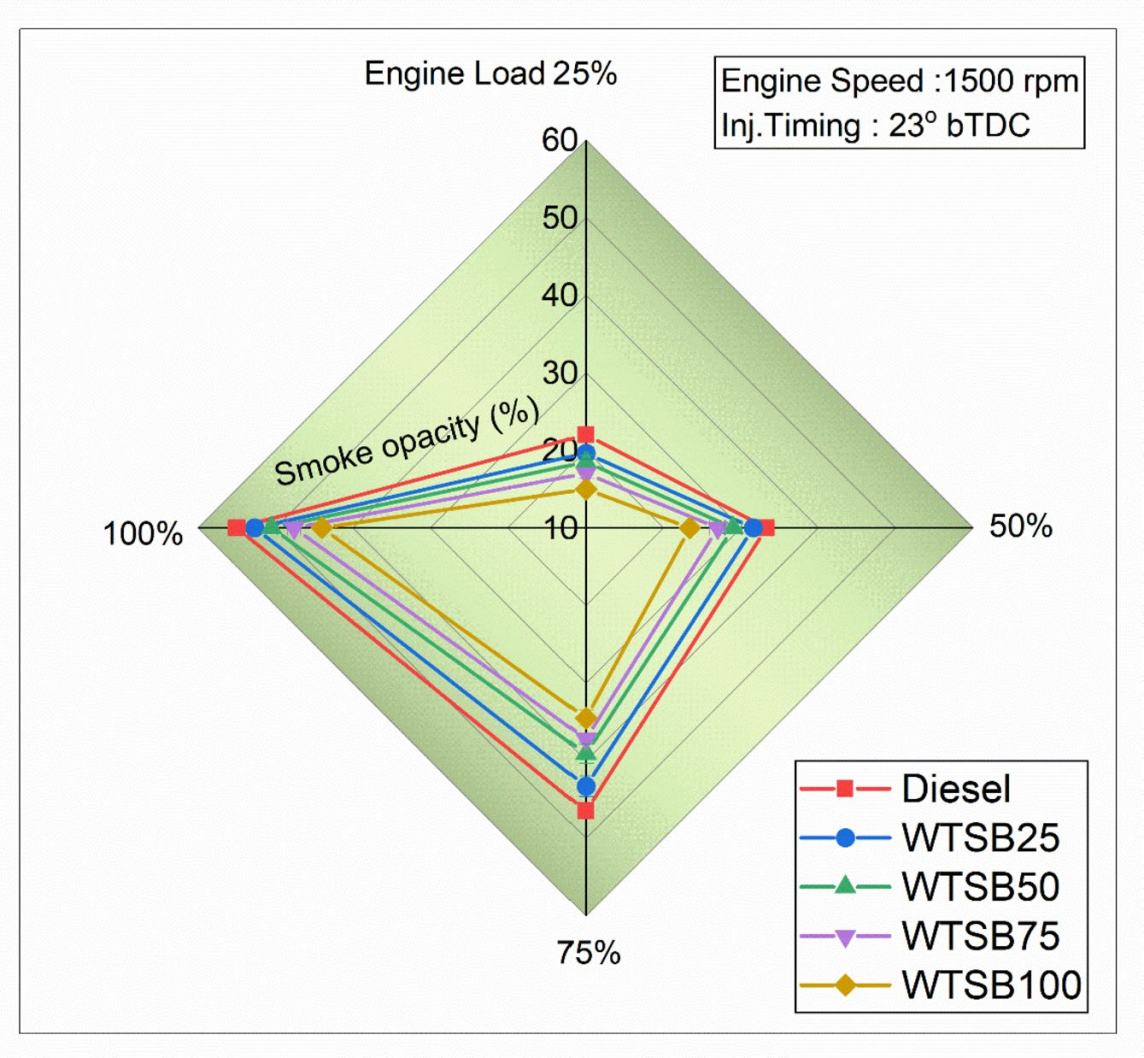
Changes in smoke opacity (%) emission for diesel and WSTB blends with engine load.

Figure 26 shows the changes in smoke opacity emissions with variable engine loads, ranging from 25 to 100%, for different fuel blends: diesel, WTSB25, WTSB50, WTSB75, and WTSB100. It was detected that smoke opacity increases gradually during DI engine operation for all WTSB blends. This occurs because, with the engine speed held constant, more fuel is injected into the cylinder as the engine load increases^76^. All WTSB blends exhibit higher smoke formation at 100% engine load compared to 25% engine load. This increase is due to the rich fuel mixtures at peak power output, leading to higher smoke emissions. However, it is notable that WTSB blends produce lower smoke emissions than existing diesel across all engine loads. This reduction may be accredited to the lower aromatic compound content, lower C/H ratio, and higher oxygen levels in green fuels, which improve combustion and fuel oxidation in fuel-rich areas^28^. At 100% engine load, smoke emissions were observed as 55% for diesel, 52.72% for WTSB25, 50.54% for WTSB50, 47.61% for WTSB75, and 44% for WTSB100. Compared to diesel fuel, smoke levels were reduced by 4.18% for WTSB25, 8.21% for WTSB50, 13.45% for WTSB75, and 20% for WTSB100. This indicates that increasing the O_2_ levels and lowering the C/H ratio in WTSB mixes effectively diminishes smoke development. Bencheikh et al.^77^ found that higher biodiesel blend proportions resulted in lower smoke levels compared to neat diesel at peak engine load. This is due to biodiesel’s oxygen content, which enhances fuel reactivity and shrinkage soot levels in the diesel exhaust.

### Comparison of present study environmental impacts with existing literature

**Fig. 27:**
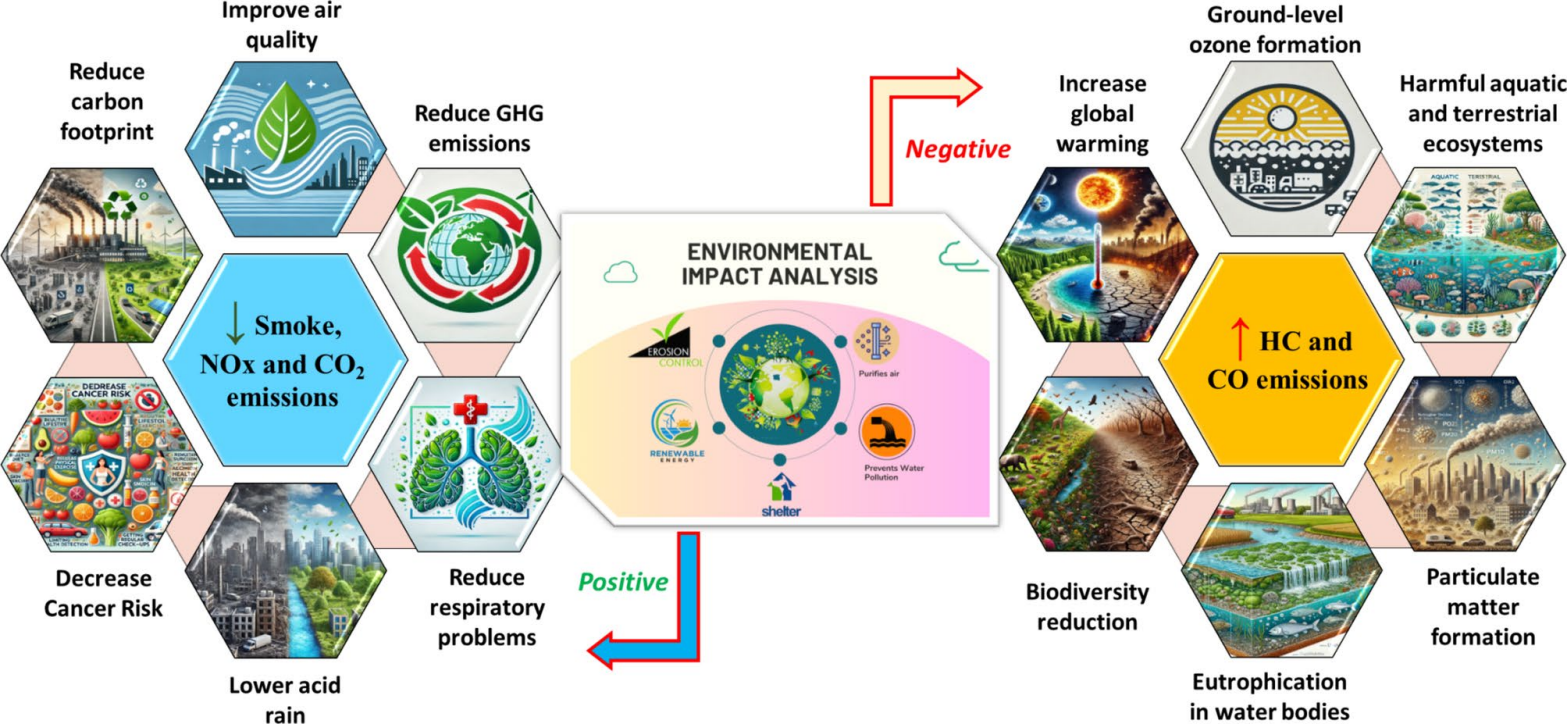
Positive and negative impacts of DI diesel engine-specific emissions on the environment.

Table 12 presents a comparison between the WTSB generated by the PJBC catalyst and the outcomes of several WTSB blends evaluated in a computerized DI diesel engine with associated environmental implications. Figure 27 shows the positive and negative effects of DI diesel engine-specific emissions on environmental impact analysis.

**Table 12:**
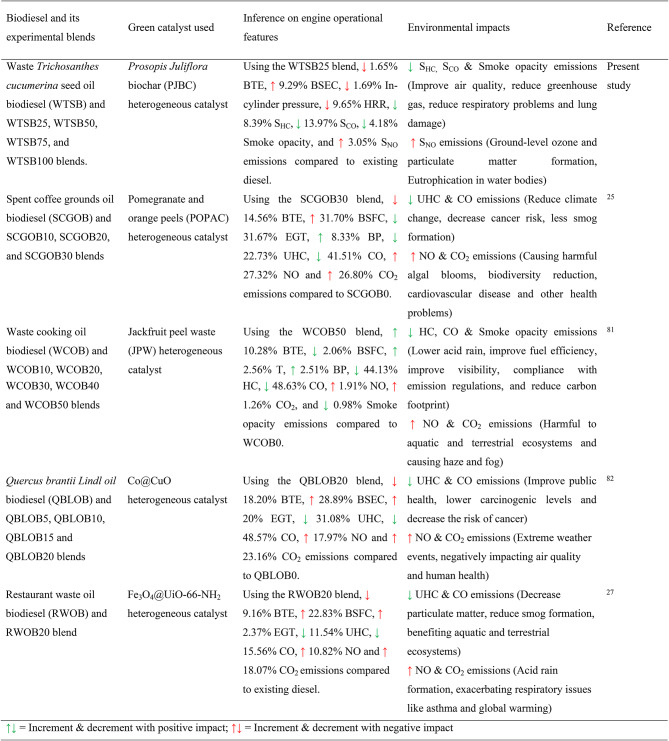
Comparing the present study’s environmental impacts with existing literature on green catalysts for biodiesel production.

### Lifecycle assessment (LCA) for the environmental impact of WTSB production and use

**Fig. 28:**
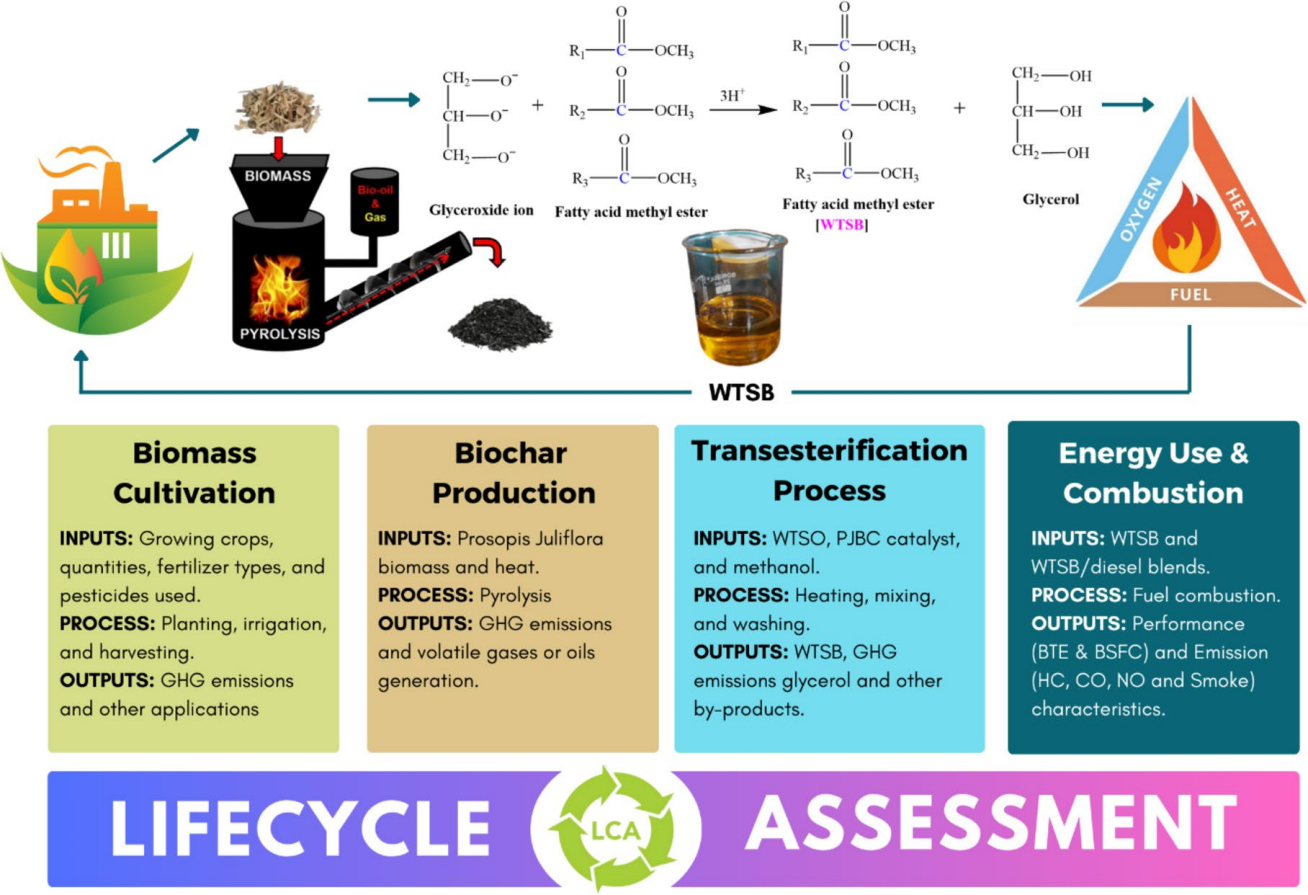
Lifecycle assessment of WTSB.

Life cycle assessment (LCA) is essential for evaluating the environmental impact of synthesized biodiesel from WTSO. The LCA method starts with the extraction of resources and goes all the way up to the creation of the final product and the disposal of wastewater. LCA systematically analyzes and quantifies the energy inputs, outputs, and environmental concerns throughout the product’s entire life cycle^80^. A comprehensive LCA of WTSB production and use encompasses key stages, including biomass cultivation, biochar production, transesterification, engine use and combustion were presented in Fig. 28.

In the cultivation stage of *Prosopis Juliflora* biomass, inputs such as water, fertilizers, pesticides, and energy for planting, irrigation, and harvesting contribute to resource utilization, while outputs include greenhouse gas (GHG) emissions from fertilizer application and machinery. Biochar production from *Prosopis Juliflora biomass* involves pyrolysis, requiring significant energy inputs and releasing GHG emissions, alongside potential by-products like volatile gases. The transesterification stage uses WTSO, PJBC catalyst, and methanol, with energy-intensive heating and mixing, yielding WTSB and by-products such as glycerol and emissions. Finally, the engine combustion stage employs WTSB and its blends in a DI diesel engine, generating specific emissions like S_HC_, S_CO_, S_NO_, and smoke opacity while assessing engine performance metrics like BTE and BSEC.

Quantifying the environmental impact of WTSB involves aggregating lifecycle GHG emissions, expressed in CO_2_ equivalents, and comparing them to existing diesel fuel. The WTSB demonstrates notable emissions and resource usage reductions, including lower energy consumption, a smaller water footprint, and reduced land use by leveraging waste feedstock and invasive species. The LCA reveals that WTSB reduces tailpipe emissions and minimizes the overall carbon footprint across cultivation, production, and use stages. This analysis underscores the sustainability of WTSB as an eco-friendly alternative to fossil fuels, showcasing its potential to contribute significantly to sustainable energy solutions while addressing environmental concerns. Incorporating these findings into research highlights the viability of WTSB as a cleaner, more resource-efficient energy source.

## Conclusions

The current study optimized the input process variables (weight, temperature, and time) to maximize the yield of PJBC using CCD-RSM in conjunction with GA. The production of WTSB with PJBC as a catalyst in the transesterification process was analyzed. Additionally, the performance of neat WTSB and WTSB/diesel blends was evaluated in a direct injection (DI) diesel engine under various load conditions. The following conclusions are drawn from the findings of this research.

- The maximum PJBC yield of 46.31% was reached with optimized input variables of 60 mg of *Prosopis Juliflora* biomass waste heated at 790 °C crucible-led tin for 60 min while using GA for optimization.
- The results of PJBC characterization investigations showed a particle shape, the presence of aromatic (C-H, bending vibration), aromatic amine (C-N, stretching vibration) and carboxylic (–COOH, stretching vibration) compounds, BCC structure with a crystallite size of 26.78 nm through SEM, FTIR and XRD analysis.
- The EDX, BET and XPS studies validated inorganic components (K, Ca, Mg, and traces of Na), specific surface area (6.281 m²/g), pore volume (0.02532 cm³/g) and average pore diameter (28.01 nm) and oxidation states on the elemental surfaces of the PJBC.
- All the characterization studies confirm that PJBC is an effective catalyst for biodiesel production, significantly enhancing the trans-esterification reaction and achieving a maximum WTSB yield of 97.42%.
- The WTSB derived from WTSO with the PJBC catalyst demonstrated excellent fuel properties of gross energy content (40.13 MJ/kg) and maximum limit of cetane rating (54) that comply with the specifications of existing diesel fuel. The highest conversion rate and functional groups of WTSB have been verified by the GC-MS and FTIR tests.
- The operation of DI diesel engine using diesel, neat WTSB, and WTSB/diesel blends demonstrates improved performance and emission outcomes. Among the WTSB/diesel blends, the WTSB25 blend achieved the highest BTE of 32.07% and the lowest BSEC of 12.25 MJ/kWh.
- Additionally, WTSB25 fuel combustion resulted in a peak in-cylinder pressure of 72.37 bar and HRR of 36.33 J/°CA, closely aligning with existing diesel fuel values.
- Emission reports indicated that neat WTSB and WTSB/diesel blends produced lower exhaust emissions across all engine load conditions. Notably, the WTSB25 blend achieved reductions in S_HC_ emissions by 8.39%, S_CO_ emissions by 13.97%, and smoke opacity by 4.18%. However, S_NO_ emissions increased by approximately 3.05% compared to diesel fuel when operated at 100% load condition.
- In conclusion, the current research indicates that optimizing input parameters will boost PJBC production rates and suggests its potential as a low-cost, eco-friendly catalyst for WTSB production. The resulting WTSB25 blend is proposed as an alternative to traditional diesel fuel for computerized DI diesel engine applications. Also, the Lifecycle assessment analysis aids in predicting and minimizing the environmental and economic impacts of WTSB production.

### Recommendations for further studies

- The future studies could focus on improving the reusability and scalability of PJBC and exploring diverse biomass feedstocks for sustainability.
- Advanced techniques like microwave-assisted pyrolysis could enhance process efficiency with high biochar yield.
- Testing WTSB blends in various engine types and mitigating nitric oxide emissions through additives or after-treatment technologies like EGR are recommended.
- Investigations can be extended to examine the characteristics of diesel engines running on WTSB/diesel blends with different fuel injection pressures and fuel injection timings.
- Integration with circular economy frameworks could further enhance resource efficiency and sustainability.

## Data Availability

The datasets used and/or analysed during the current study available from the corresponding author on reasonable request.

## Abbreviations

ANOVA: Analysis of variance
ASTM: American Society for Testing and Materials
BTE: Brake thermal Efficiency
BET: Brunauer–Emmett–Teller
BSEC: Brake specific energy consumption
CCD: Central composite design
DI: Direct injection
EDX: Energy-dispersive X-ray spectroscopy
FTIR: Fourier transform infrared spectroscopy
GC–MS: Gas chromatography-mass spectrometry
GA: Genetic algorithm
HRR: Heat release rate
LCA: Lifecycle assessment
PJBC: *Prosopis Juliflora* Biochar
RSM: Response surface methodology
S_HC_: Specific hydrocarbon emission
S_CO_: Specific carbon monoxide emission
S_NO_: Specific nitric oxide emission
SEM: Scanning electron microscope
WTSO: Waste *Trichosanthes cucumerina* seed bio-oil
WTSB25: 25% WTSB + 75% diesel fuel
WTSB50: 50% WTSB + 50% diesel fuel
WTSB75: 75% WTSB + 25% diesel fuel
WTSB100: 100% WTSB + 0% diesel fuel
WTSB: Waste *Trichosanthes cucumerina* seed biodiesel
XPS: X-ray photoelectron spectroscopy
XRD: X-ray diffraction
